# Identification of Volatiles of the Dinoflagellate *Prorocentrum cordatum*

**DOI:** 10.3390/md20060371

**Published:** 2022-05-30

**Authors:** Diana Koteska, Selene Sanchez Garcia, Irene Wagner-Döbler, Stefan Schulz

**Affiliations:** 1 Institute of Organic Chemistry, Technische Universität Braunschweig, 38106 Braunschweig, Germany; d.koteska@tu-braunschweig.de; 2 Institute of Microbiology, Technische Universität Braunschweig, 38106 Braunschweig, Germany; selene.sanchezgarcia@helmholtz-hzi.de (S.S.G.); irene.wagner-doebler@helmholtz-hzi.de (I.W.-D.)

**Keywords:** VOC, headspace analysis, OSSA, thermodesorption, GC/MS, algal volatiles

## Abstract

The dinoflagellate *Prorocentrum cordatum*, often called *P. minimum*, is a potentially toxic alga found in algal blooms. Volatile compounds released by the alga might carry important information, e.g., on its physiological state, and may act as chemical messengers. We report here the identification of volatile organic compounds emitted by two strains, xenic *P. cordatum* CCMP 1529 and axenic *P. cordatum* CCMP 1329. The volatiles released during culture were identified despite their low production rates, using sensitive methods such as open-system-stripping analysis (OSSA) on Tenax TA desorption tubes, thermodesorption, cryofocusing and GC/MS-analysis. The analyses revealed 16 compounds released from the xenic strain and 52 compounds from the axenic strain. The majority of compounds were apocarotenoids, aromatic compounds and small oxylipins, but new natural products such as 3,7-dimethyl-4-octanolide were also identified and synthesized. The large difference of compound composition between xenic and axenic algae will be discussed.

## 1. Introduction

*Prorocentrum cordatum*, previously described as *P. minimum* [1], is a widespread, bloom-forming, photosynthetic dinoflagellate that causes harmful algal blooms, red tides, in many coastal estuarine ecosystems around the world [2,3,4,5]. This alga has been reported to have toxic effects on humans, leading to poisoning or even death by ingesting foods such as shellfish [6,7,8], oysters [9] or clams [10,11,12,13], although these claims have been recently challenged [1]. In addition, the dinoflagellate can also have harmful effects on ecosystems and organisms, causing environmental damages resulting from huge algal biomass [3], pH change [3], striking light attenuation in bloom-forming regions [3] and oxygen depletion causing fish [14,15,16] as well as zoobenthos death [16]. Because of its geographical expansion, availability in many areas and easy culturing, the alga has become an important research object [15] and developed into a model system for studying, e.g., the interaction of microalgae and bacteria [17,18]. Earlier studies focused on the extracellular secondary metabolites of *P. cordatum,* as these metabolites are thought to play an important role in interactions between phytoplankton and other organisms. Such a metabolite is 1-(2,6,6-trimethyl-4-hydroxycyclohexenyl)-1,3-butanedione, a non-volatile apocarotenoid identified in filtrates of laboratory cultures of *P. cordatum* (as *P. minimum*) [19,20,21]. This compound seems to be a degradation product of peridinin, the major carotenoid of *P. cordatum* [22]. Apocarotenoids are degradation products of carotenoids such as β-carotene, e.g., obtained from their photo-oxygenation or from enzymatic degradation [23,24].

Microbial volatiles have recently been recognized to be important mediators of organismic interactions [25]. Although seemingly contradictory, volatiles are not only perceived in the gas phase but are also important in water due to their fast diffusion, thereby serving as the first chemical cues present when organisms come into closer contact [25]. Therefore, we assume that the composition of the algal volatile organic compound (VOC) bouquet might transport information on the physiological state of the emitter and its identity due to potentially specific volatile mixtures, which might be recognized by other micro- or macro-organisms. Furthermore, these volatiles might also serve as signals, mediating interactions, e.g., between algae and bacteria [26]. Differences between the growth of *P. cordatum* under xenic and axenic conditions due to different physiology and metabolism have been observed [27,28]. An analysis of the volatiles released might therefore shed light on the physiological status of *P. cordatum*.

*P. cordatum* has been previously analyzed for the production of volatile unsaturated aldehydes, but no dominant volatiles were found, neither by undisturbed cultures of the alga nor after its wounding. It was concluded that a wound-activated defense by the transformation of fatty acids into volatile aldehydes is lacking in this alga in contrast to other algal species [29].

Because algae grow slowly compared to many bacteria and their cell densities in cultures are much smaller, the release rate of volatiles from many algae is low. Therefore, a sensitive method using headspace desorption with GC/MS was used to detect and identify the volatile compounds. Investigations of the volatilome of xenic algae and their axenic counterparts are rare, and little is known about the function VOCs in these algae.

The aim of the study was to chemically identify the volatiles released from a xenic and an axenic strain, to identify similarities as well as differences in the bouquet composition, and detect volatiles potentially specific for *P. cordatum*. In addition, this approach could provide insight into volatiles originating from the algae and associated bacteria or are a consequence of the interaction of the bacteria with their algal host. Surprisingly, the axenic strain *P. cordatum* CCMP 1329 produced more compounds than the xenic *P. cordatum* CCMP 1529 strain, although volatiles emitted by the accompanying bacteria would be expected to contribute to the bouquet in case of the latter strain.

The axenic *P. cordatum* strain CCMP 1329 has been studied as a model host for the roseobacter-group bacterium *Dinoroseobacter shibae*, and it could be shown that the bacteria can provide vitamins to the dinoflagellate but kill the algae during later stages of growth [17]. Killing may be the result of competition between the alga and bacterium for biotin, a vitamin that is essential for both organisms [30]. The microbiome associated to strains of *P. cordatum* maintained in culture collections, e.g., CCMP 1529, differs according to the biogeographical region and time since isolation (Sanchez-Garcia, in preparation). In natural algal blooms, roseobacter-group bacteria have been shown to be an important part of the microbial community, together with other Alphaproteobacteria, Gammaproteobacteria and Bacteroidetes [31].

## 2. Results

### 2.1. Volatile Analysis

**Figure 1:**
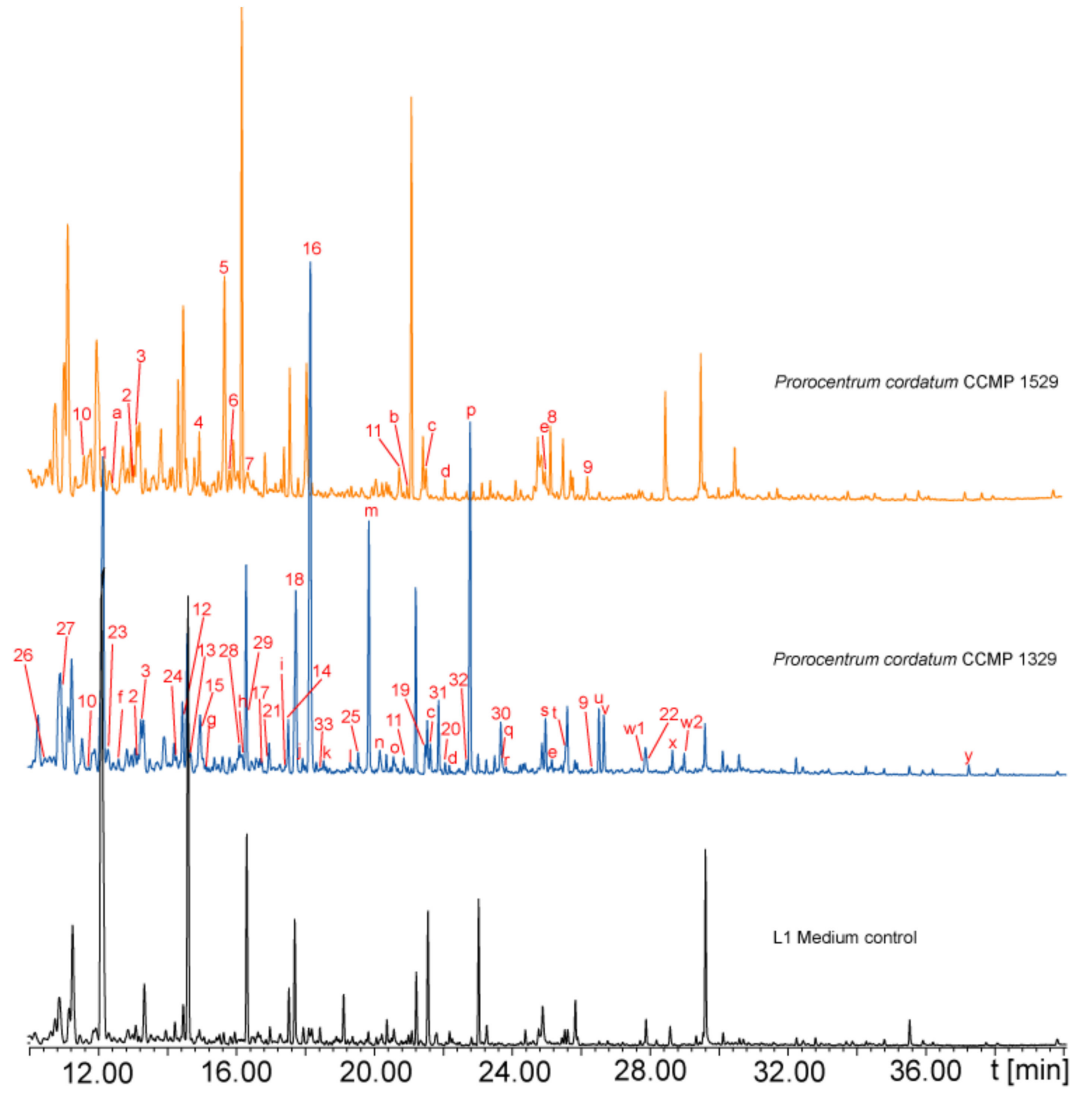
Total ion chromatogram (TIC) of typical OSSA headspace analyses of xenic *Prorocentrum cordatum* CCMP 1529, axenic *P. cordatum* CCMP 1329 and the L1 medium control. The numbers in the chromatograms refer to the identified compounds shown in Table 1 and Table 2. Unlabeled peaks originate from the medium.

The detection of VOCs by the GC/MS of living algal cultures is often difficult due to the low emission rates of volatile compounds. Typical dynamic headspace collection methods such as the closed-loop-stripping analysis (CLSA) [32,33] need long capturing times and have a comparatively low sensitivity because only a small fraction of an extract is transferred to the GC/MS system for analysis. In contrast, the dynamic trapping of the VOCs on adsorbents followed by thermodesorption into the GC/MS system allows for a more sensitive analysis [32]. In this study, the headspace collection was performed using an open-system-stripping analysis (OSSA) with Tenax TA desorption tubes, followed by thermodesorption, cryofocusing and the GC/MS analyses of liquid cultures of the alga *P. cordatum* during the stationary phase. The method combines relatively short extraction times of 1–4 h and a high sensitivity due to the injection of the whole collected material into the GC/MS instrument. Solvents are not necessary and even highly volatile compounds can be analyzed. The analyses were performed on the axenic *P. cordatum* CCMP 1329 and xenic *P. cordatum* CCMP 1529 cultures, each with three biological replicates, as well as the respective medium control samples (Figure 1). The medium also proved to release volatiles, mostly hydrocarbons, which have been reported as algal constituents by others, but which are obviously contaminations [34,35], complicating the analyses. Nevertheless, due to the good chromatographic separation, compounds originating from the algae were clearly detectable because they were absent in the medium control. For the identification of compounds, mass spectra and retention indices from the NIST mass spectral library and our own in-house database were used. In the case of unknown mass spectra or retention indices, commercially available standards were used, or syntheses of candidate structures were performed as described in the next sections.

### 2.2. Identification of Algal Volatiles

**Figure 2:**
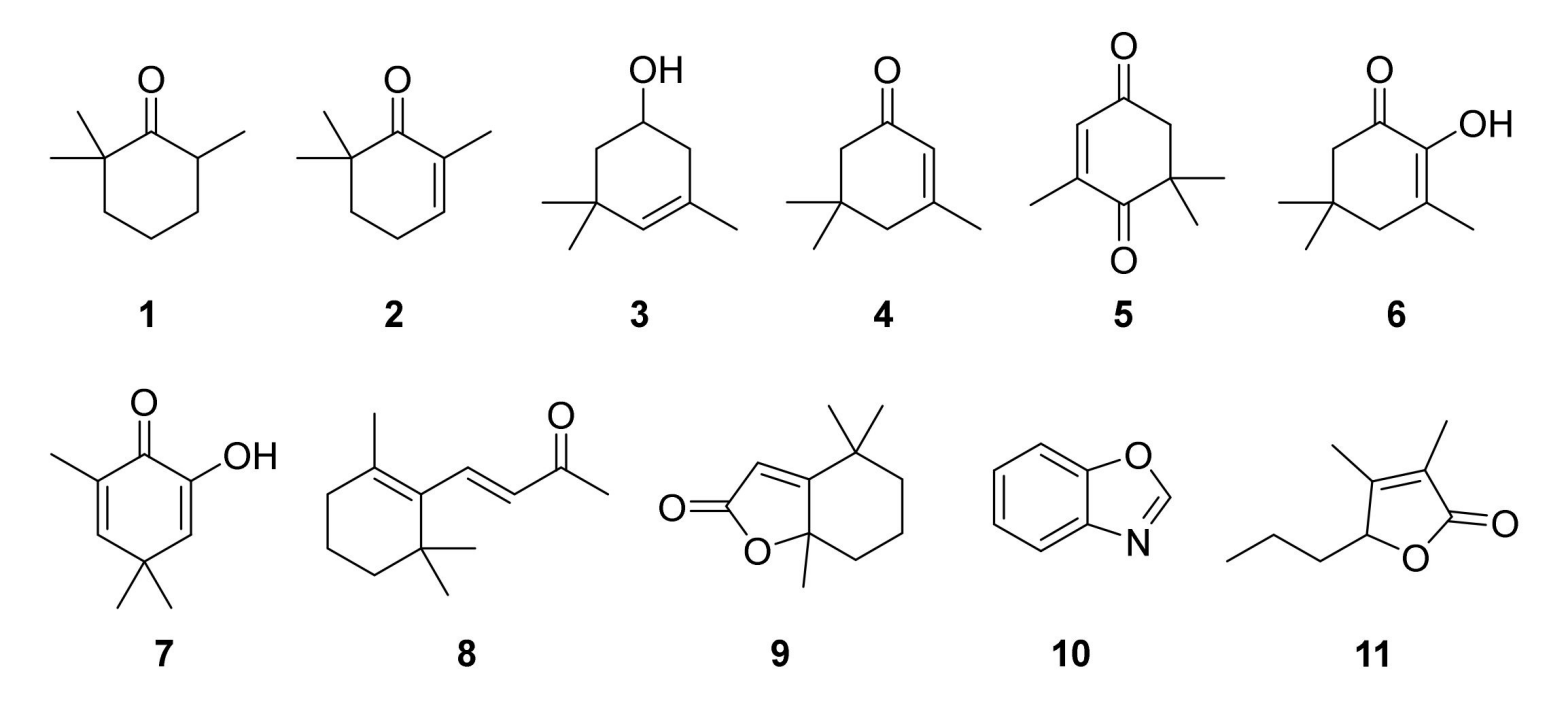
Volatiles from the xenic strain *Prorocentrum cordatum* CCMP 1529.

The analysis of xenic *P. cordatum* CCMP 1529 revealed only a comparatively low number of VOCs, shown in Figure 2 and Table 1. There were 16 known and unknown compounds released from all three replicates. All other peaks could be assigned to the background. Besides some unknown compounds, the largest group of volatiles were apocarotenoids (**1**–**9**), but also benzoxazole (**10**) and the unsaturated lactone **11** were identified. 

**Figure 3:**
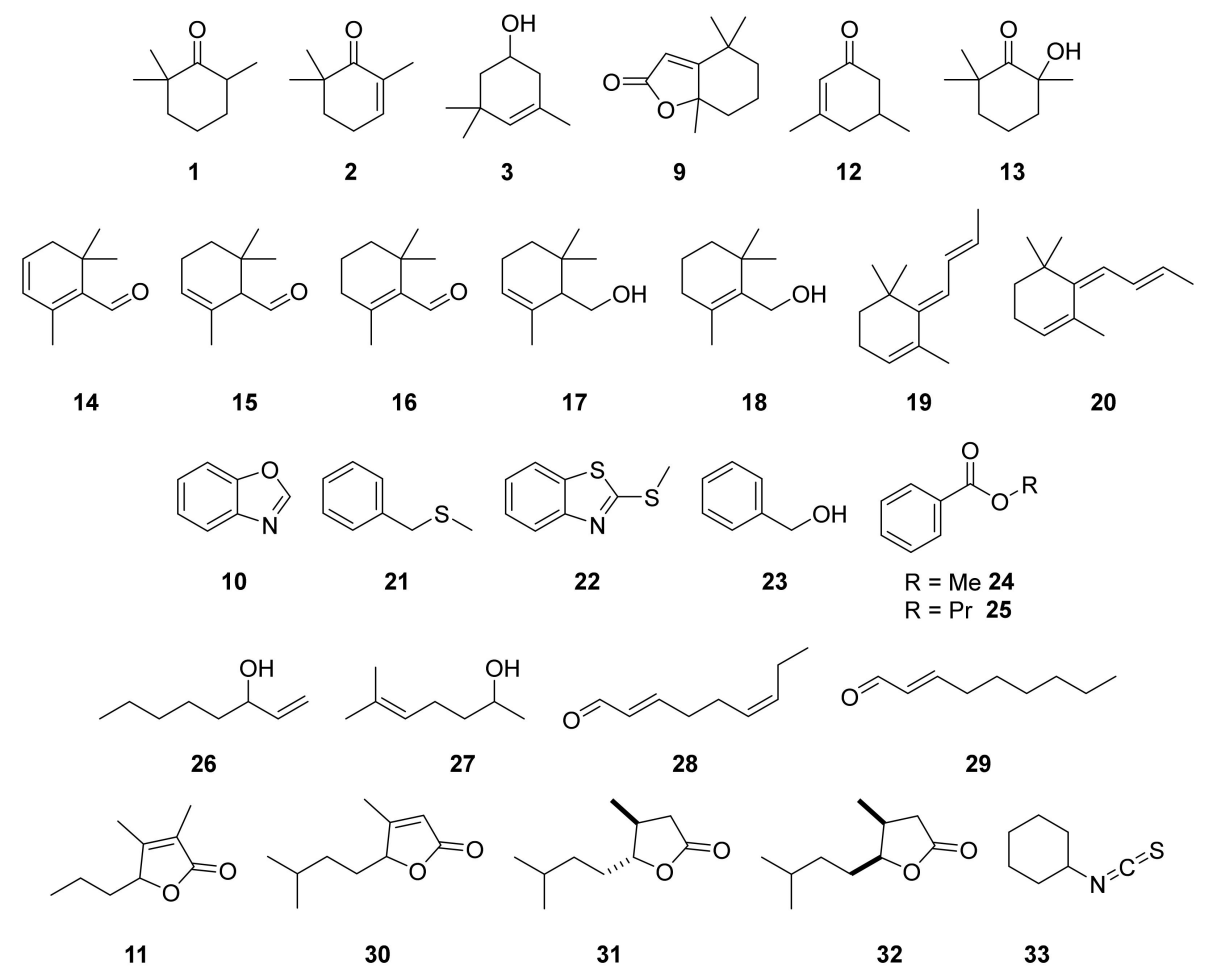
Volatiles from the axenic strain *Prorocentrum cordatum* CCMP 1329.

The headspace extracts of the axenic strain *P. cordatum* 1329 comprised more volatiles compared to the xenic strain (Figure 3 and Table 2). Within the 52 known and unknown compounds, apocarotenoids were again the largest group of emitted VOCs, accompanied by aromatic compounds and aliphatic compounds comprising unsaturated alcohols and aldehydes, lactones, as well as cyclohexyl isothiocyanate (**33**). The apocarotenoid β-cyclocitral (**16**) was the most abundant constituent of all three replicates, followed by α-cyclocitral (**15**) and β-cyclogeraniol (**18**). 6-Methyl-5-hepten-2-ol (**27**) and 3,5,5-trimethylcyclohex-3-en-1-ol (**3**) were also abundant in some replicates. 

**Scheme 1:**
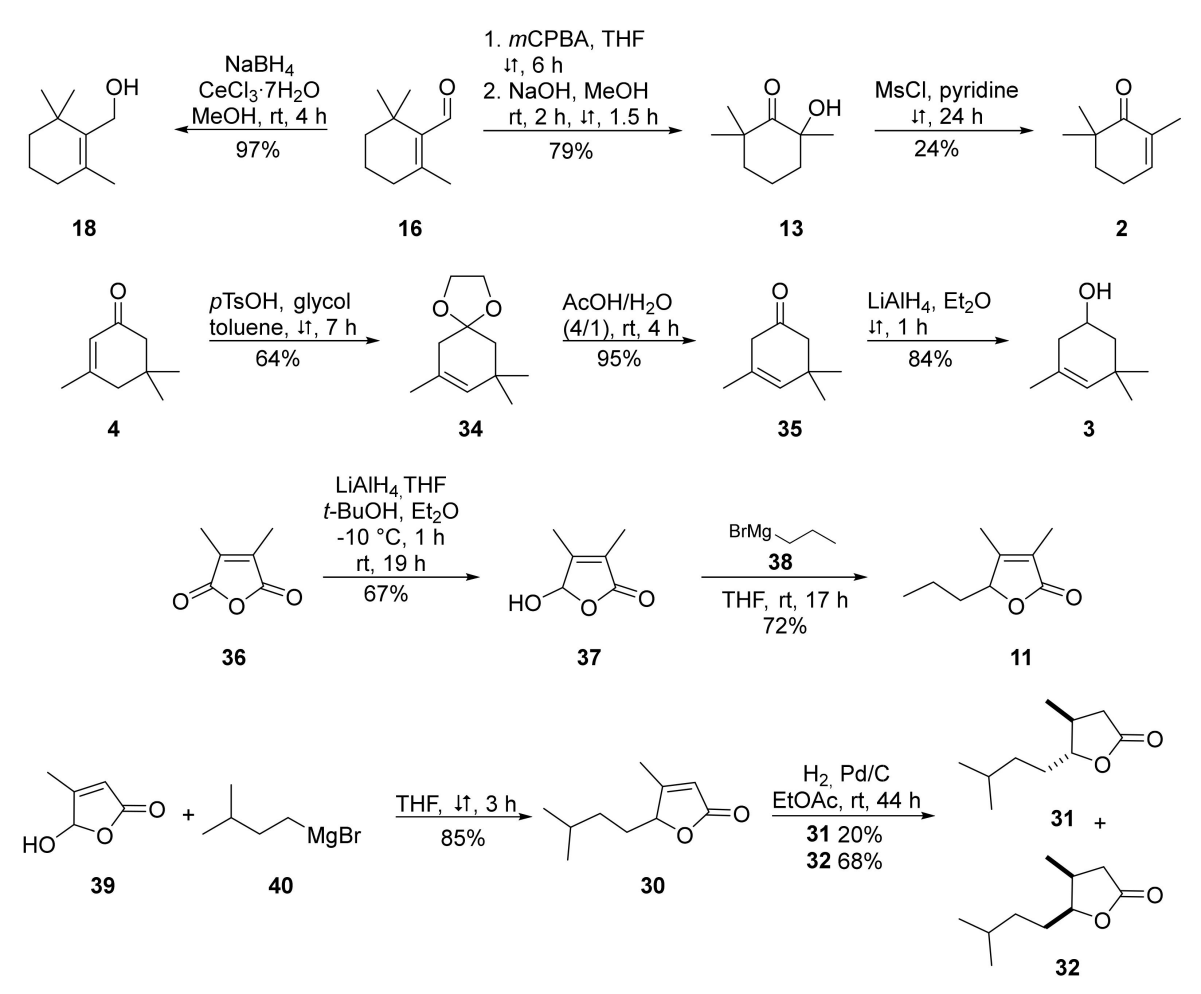
Syntheses of the reference compounds **2**, **3**, **11**, **13**, **18**, **30**, **31** and **32**.

Although many compounds were identified by mass spectral data and gas chromatographic retention indices (*I*), some needed additional comparison with authentic samples that were synthesized (Scheme 1). The reported *I*-value for 2,6,6-trimethylcyclohex-2-en-1-one (**2**) [36] differed from our experimental value. Therefore, compound **2** was synthesized from β-cyclocitral (**16**). Oxidation of **16** with *m*CPBA and hydrolysis yielded 2-hydroxy-2,6,6-trimethylcyclohexan-1-one (**13**) [37], another algal constituent. Final elimination with mesyl chloride [38] gave compound **2**, identical in all aspects to the natural compound, confirming its identification. Likewise, 3,5,5-trimethylcyclohex-3-en-1-ol (**3**) was obtained by the deconjugation of α-isophorone (**4**) and its *I*-value was confirmed. The acetalization of **4** with glycol leads to the rearrangement of the double bond in **34**. This acetal was deprotected using acetic acid to give compound **35** [39]. The subsequent reduction with LiAlH_4_ yielded the β,γ-unsaturated alcohol **3** [40]. An unknown compound with a mass spectrum similar to that of α-cyclogeraniol (**17**) was identified as β-cyclogeraniol (**18**), obtained by the Luche reduction of **16** [41].

In addition to these apocarotenoids, several lactones were identified. Compound **11** showed a mass spectrum (Figure S1 in the SI) similar to that of the known 2,3-dimethyl-2-nonen-4-olide but lacking a C_2_H_4_ unit. Therefore, 2,3-dimethyl-2-hepten-4-olide (**11**) was synthesized by converting 3,4-dimethylfuran-2,5-dione (**36**) with LiAlH_4_ into 5-hydroxy-3,4-dimethylfuran-2(5*H*)-one (**37**), the starting material for the following Grignard reaction with propylmagnesium bromide (**38**) [42,43]. The product **11** proved to be identical with the natural compound. 

The unknown compounds **31** and **32** showed very similar mass spectra (Figure S1), whose closest library match was δ-undecalactone. The presence of two obvious diastereomers indicated at least two stereogenic centers. In addition, the retention index was about 150–180 units lower than that of δ-undecalactone. Therefore, a C-2 or C-3 methyl-substituted γ-lactone with an isopentyl side chain at C-4 was proposed as the structure, sharing the typical *m*/*z* 99 ion with δ-lactones in the mass spectra. The 3-methyl compound seemed to be more likely, because the structure would fit a terpene carbon skeleton. For the synthesis, 5-hydroxy-4-methylfuran-2(5*H*)-one (**39**) was reacted with isopentyl magnesium bromide (**40**) to give the unsaturated lactone **30**. The hydrogenation of **30** furnished the diastereomers **31** and **32** [44] that were separated and their structures assigned, via literature NMR data of related lactones (see Supporting Information), to be *trans*- and *cis*-3,7-dimethyl-4-octanolide. Moreover, the mass spectrum and retention index of the synthetic intermediate **30** coincidentally matched the data of another unknown constituent, thus confirming its structure to be 3,7-dimethyl-2-octen-4-olide.

## 3. Discussion

In the following section, the different compound classes will be discussed in view of the compound occurrence in algae and biological functions, followed by a more general discussion.

### 3.1. Apocarotenoids

Apocarotenoids comprised the largest group of VOCs from both strains. Being carotenoid degradation products, apocarotenoids are typically found as algal volatiles. Common to both strains were 2,2,6-trimethylcyclohexan-1-one (**1**), 2,6,6-trimethylcyclohex-2-en-1-one (**2**), 3,5,5-trimethylcyclohex-3-en-1-ol (**3**) and dihydroactinidiolide (**9**). The apocarotenoids **4**–**8** were specific for the xenic strain, whereas compounds **12**–**20** were only detected in the axenic strain. Such apocarotenoids are oxidative degradation products of carotenoid pigments that harvest light energy and protect the algae from photo-oxidation. The carotenoid cleavage to the smaller VOCs can be induced enzymatically by dioxygenases or non-enzymatically by reactive oxygen species (ROS) [45]. Oxidation of carotenoids lowers the oxidative damage in algae, resulting in higher release rates of apocarotenoid VOCs. Thus, damage to the photosynthetic apparatus, to cell membranes or even the induction of programmed cell death can be avoided [46]. 

The apocarotenoid VOCs of *P. cordatum* are derived from its carotenoids. While peridinin has been reported as the major carotenoid in this group of dinoflagellates [22], the occurrence of other carotenoids is also likely (β-carotene, fucoxanthin and others) [47]. Interestingly, carotene degradation would result in apocarotenoids carrying a typical 2,2,6-trimethylcyclohexyl structural element (isophorone type) as observed in the C_9_-, C_10_- and C_13_-apocarotenoids reported here [46,48,49,50,51,52]. In contrast, a 4-hydroxy-2,2,6-trimethylcyclohexyl element, obtained from peridinin or fucoxanthin degradation, was less often observed (**3**–**7**), preferentially in the xenic strain. Nevertheless, several of the unidentified compounds might also contain this structural element. The C_9_-norisoprenoid 4-oxoisophorone (**5**) carrying the latter structural element was described to be formed from violaxanthin and zeaxanthin carotenoids, not reported from *P. cordatum* so far [49]. 

Several of the apocarotenoids are known from other algae. Ketone **1** is a VOC occurring in the essential oil of the green alga *Capsosiphon fulvescens* [53] and the marine green macroalga *Ulva lactuca* [54], and also in monocultures of the xenic alga *Scenedesmus subspicatus*, which was one of the algae responsible for the musty tastes and odors of drinking water supplies [55]. The unsaturated isomer **2** has only been reported from mixtures of algae and different cyanobacteria [56]. Alcohol **3** was identified in the essential oil of *U. lactuca* [54]. α-Isophorone (**4**) is commonly found in the essential oils of diverse plants [57,58,59,60,61] and also in the essential oil of *U. lactuca* [54]. Its hydroxy-derivative 2-hydroxyisophorone (**6**) was reported from the hydrodistillate of the air-dried green alga *Codium adhaerens* [62]. 2-Hydroxy-4,4,6-trimethylcyclohexa-2,5-dien-1-one (**7**) has not been reported from algae so far. 4-Oxoisophorone (**5**), *trans*-β-ionone (**8**) and dihydroactinidiolide (**9**) are likewise widespread apocarotenoids [53,55,62]. Diketone **5** was recently found in the essential oils of the algae *Cystoseira tamariscifolia*, *Sargassum muticum* and *U. lactuca* [54], while **8** and **9** were present in the Japanese macroalgae *U. prolifera*, *U. linza* and *Monostroma nitidum* [49,63]. 

α- and β-cyclocitral (**15** and **16**) were identified here together with their respective alcohols, α- and β-cyclogeraniol (**17** and **18**). *trans*-β-Ionone (**8**) and **16** are known as the dominating volatiles in algal and cyanobacterial bloom periods [64], affecting water quality by causing odor in water supplies [65]. β-Cyclocitral (**16**) showed lytic activity against cyanobacteria [66] and causes color change in the cyanobacteria cultures from green to blue, as this is observed at Lake Tsukui in Japan [67]. Compounds **8** and **16** are also allelopathic, as they inhibited the cell growth of the green alga *Chlorella pyrenoidosa* [68]. Compound **16** caused cell rupture in *Microcystis aeruginosa* and another diatom, *Nitzschia palea* [69], while **8** reduced the growth of *Enteromorpha compressa* and *Lemna pausicostata* [70]. Compound **16** has also been observed in the wild, being the major component, accompanied by **15, 8**, **1** and **2**, in water from a eutrophic shallow lake. This lake contained different species of dominant algae and cyanobacteria, such as the *Microcystis wesenbergii*, *Oscillatoria redekei*, *Diatoma* or *Chlorophycae* species [71]. Compound **15** was also observed in the air-dried hydrodistillate of *C. adhaerens* [62]. Cyclogeraniols **17** and **18** are known from diverse plants [72,73,74,75,76] and cyanobacteria [77,78]; both were not reported from algae so far. Safranal (**14**), a common carotene-derived aroma compound [79,80], is best known for its occurrence as the main flavor of *Crocus sativus* [61,81,82]. Recently, it was reported as a component in the essential oils of the brown algae *Dictyopteris polypodioides* [83], *C. tamariscifolia* and *U. lactuca* [53]. 3,5-Dimethylcyclohex-2-en-1-one (**12**) is probably not an apocarotenoid because of its missing methyl group. It is not known from algae, but has been detected as a fungal volatile [84]. The hydroxy ketone **13** was reported from the headspace of *U. prolifera* and *U. linza* [63], as well as from the blue-green algae *Phormidium* and *Rivularia* [78].

The megastigmatrienes **19** and **20** are for the first time reported from algae and represent with **8** the only C_13_-apocarotenoids observed. To the best of our knowledge, 6-methylhept-5-en-2-ol (**27**) is known from diverse plants [85,86,87] but not from algae. It might be formed via the apocarotenoid pathway from an open chain precursor but, alternatively, also by the degradation of other open chain terpenes. 

### 3.2. Aromatic Compounds

Another group of VOCs are aromatic compounds, likely obtained via the shikimate pathway [50,88] that has been reported from dinoflagellates, producing mycosporine-like amino acids [89,90,91] with various protective functions in marine organisms [92,93,94]. Again, the difference between the xenic and axenic conditions is large, with only benzoxazole (**10**) present in both strains in low concentrations, while other aromatic compounds occur only in the axenic strain as minor components. Benzoxazole (**10**) has been identified from the bio-oil of the green microalga *Scenedesmus obliquus* after hydrothermal liquefaction [95]. Benzyl alcohol (**23**) is a widespread VOC among bacteria [50] and has been reported from the brown algae *Padina pavonia* and *C. adhaerens* [62,96]. To the best of our knowledge, the only sulfide identified, benzyl(methyl)sulfane (**21**), as well as methyl and propyl benzoates (**24**, **25**) and the benzothiazol derivative (**22**) have not been reported from algae. Nevertheless, it is a known degradation product of the biocide 2-(thiocyanomethylthio)benzothiazole (TCB) [97]. Therefore, its identification should be regarded as tentative, although we are not aware of the use of TCB in any part of our experiments.

### 3.3. Aliphatic Compounds

The unsaturated lactone 2,3-dimethyl-2-hepten-4-olide (**11**) was the only aliphatic compound released from both *P. cordatum* strains. Although it is so far unknown from algae, it was already reported as volatile flavor compound of dried bonito [98]. The structure resembles those of common furan fatty acids which are produced by lipoxygenases from unsaturated fatty acids [99]. In the end, this compound might be a final product of acid lipoxygenation. The lactone functional group is a typical structural motif found in signaling compounds [100] and also in algae [101]. The γ-lactones **30**–**32** have not been reported as natural products so far but share some structural features with the A-factor-type signaling compounds of *Streptomyces* bacteria [102]. Considering their carbon framework, these lactones can be qualified as monoterpenes and might therefore be related with the apocarotenoids discussed above, although no immediate biosynthetic link is obvious. Similar γ-lactones of the roseobacter-group bacterium *Ruegeria pomeroyi* have been shown to have algicidal properties in high concentration [103].

The aldehydes (2*E*,6*Z*)-2,6-nonadienal (**28**) and (*E*)-2-nonenal (**29**) are oxylipins derived from polyunsaturated fatty acids (PUFAs). In higher plants, they are formed in response to abiotic or biotic stress in defense [104] and serve as messengers for communication with nearby plants [105]. Such aldehydes are also involved in the chemical defense of the alga *Thalassiosira rotula* which, after cell disruption, forms unsaturated aldehydes of a similar chain length [106]. The same mechanism was observed in the macroalgae *U. rigida* and *U. ohnoi* upon wounding [107]. Boonprab et al. proposed an oxylipin pathway to the major component (*E*)-2-nonenal (**29**) in the brown alga *Laminaria angustata* starting from arachidonic acid (PUFA) [108,109]. Aldehydes **28** and **29** were also reported from *S. subspicatus* [54] and from the essential oil of the green alga *C. fulvescens* [53]. Another well-known oxylipin is 1-octen-3-ol (**26**), a widespread fungal aroma constituent [110,111], also reported in the alga from *C.* *adhaerens* [62]. In the alga *Pyropia haitanensis,* arachidonic acid is oxidatively cleaved by lipoxygenases, forming **26** and **29** [112]. Alcohol **26** acts as an oxylipin messenger inducing a primed state of *P. haitanensis* upregulating the synthesis of other signaling compounds such as indole-3-acetic acid or methyl jasmonate. A concentration-dependent inhibition of the decay of the algae and a reduced number of epiphytic bacteria on it were observed [113]. Thus, it could act as an elicitor inducing *P. haitanensis* resistance. Volatile oxylipins were also increasingly formed after treatment with **26** [113]. Finally, the unique compound cyclohexyl isothiocyanate (**33**) has been reported earlier from the Black Sea red alga *Bangia fuscopurpurea* [114]. All compounds **26**–**33** occurred only in the axenic strain.

### 3.4. Function of the Identified Volatiles

Natural VOCs have important functions [25] and their emission is affected by environmental and ecological factors, including light, temperature, nutrition conditions, abiotic stress and others [46]. Moreover, to resist biotic stress, VOCs can be emitted to induce defense responses against predators or pathogens in aquatic systems or prime cells for a stress response [46,115].

The comparison of the VOC bouquet revealed a large difference between the two strains, with the axenic strain producing a markedly larger number of compounds in a higher concentration. The influence of symbiotic bacteria on the volatile bouquet due to the direct release of volatiles seems to be low. Apocarotenoids **1**–**3** and **9**, benzoxazole (**10**) and the lactone **11** were produced by both strains, confirming their formation by the algae. This cannot be said with certainty for the apocarotenoids **4**–**8**, as they were specific for the xenic strain. Nevertheless, apocarotenoids are relatively rarely observed as volatiles of bacteria except for cyanobacteria [50,116]. In contrast, algae seem to readily release apocarotenoids as discussed here.

The large difference in the number of compounds, including several still unidentified, mostly minor components as well as the formation of the dominating compounds **15, 16** and **18,** may be a direct consequence of missing bacteria in the strain *P. cordatum* CCMP 1329. It has been shown that *P. cordatum* growing symbiotically with the roseobacter-group bacterium *Dinoroseobacter shibae* obtains vitamins B_1_ and B_12_ from the bacterium [17,30]. The medium of the axenic strain did contain these vitamins, thus excluding the possibility that the increased VOC production is a direct consequence of a lack of vitamins. In addition, the axenic strain showed improved growth compared to the xenic strain (Sanchez-Garcia et al., in prep.). In addition to vitamin transfer, symbiotic bacteria of algae can, among other tasks, take up primary metabolites from algae as dissolved organic matter (DOM) and supply inorganic nutrients [117,118,119]. It might therefore be that part of the VOCs are taken up by the bacteria in the culture as DOM [120,121], as has been shown, e.g., for the volatile isoprene [122,123]. In addition, compounds released by the bacteria and taken up by mixotrophic *P. cordatum* may influence the algal VOC production. Such an uptake may influence the algal physiology, which leads to an altered VOC emission. For example, the roseobacter-group bacterium *Ruegeria pomeroyi* triggered differential expression of over 80 genes in the diatom *Thalassiosira pseudonana* [26].

The large number of apocarotenoids, aromatic compounds and oxylipins of *P. cordatum* may also act as signals or compounds influencing surrounding micro- or macro-organisms. As described above, several of the compounds have already been proven to have, e.g., allelopathic effects and may be actively formed as a defense mechanism, e.g., **8**, **16** and **26.** Especially the PUFA-derived oxylipins of aldehydes and alcohols **26**–**29** can be involved in the chemical defense in algae [124]. For the unique lactones **31** and **32** as well as compounds **11** and **30**, no ecological function is known, but due to their specificity, a function as signaling compounds of *P. cordatum* is plausible.

In summary, the observed VOC bouquet may be exploited as a general signal of the physiological state of the algae. Although we are not aware of any inter- or intraspecific interaction of such signals, they can potentially be exploited to transfer information, may it beneficial or detrimental for *P. cordatum*. Apart from the discussed possible biological activities of the algae, the bouquet itself may also serve as a species indicator, because a species-specific bouquet is formed. In this bouquet non-specific compounds such as, e.g., **4**, **9** or **23**, more class-specific compounds such as apocarotenoids and also specific compounds such as **31** and **32** are included. These classes of compound occurrence have been recently defined to structure VOC occurrence in microorganisms [25].

## 4. Materials and Methods

### 4.1. Strains and Culture Conditions

Xenic *Prorocentrum cordatum* CCMP 1529 and axenic *P. cordatum* CCMP 1329 strains were obtained from Bigelow National Center for Marine Algae and Microbiota (NCMA). Both strains were cultivated in L1 Medium according to the recipe of Guillard and Hargraves [125] in synthetic ocean water [126], but Na_2_SiO_3_·9H_2_O was omitted because *P. cordatum* does not need it. The strains were cultivated in RuMed incubators set to 26 °C and were grown in 100 mL batches in 300 mL Erlenmeyer flasks under a 12:12 h light–dark cycle with a light intensity of about 40 μmol photons m^−2^s^−1^. *P. cordatum* cultures were maintained by transferring 10% of the culture at the late exponential phase to fresh medium every 10 days. Algae cultures were maintained at 26 °C for at least 4 growth periods before samples for MiSeq sequencing were collected. All experimental work and algae transfers were performed under a laminar flow hood using sterile conditions. Growth of algae was followed by cell counting using a BD FACS Canto flow cytometer (BD Biosciences, San Jose, CA, USA), according to the methods described previously [18]. The axenic strain was checked for lack of contaminating bacteria by streaking aliquots on marine agar (MB) medium plates. For the headspace analyses, the strains were cultivated at 26 °C for 19 days at the same light–dark cycles and the same light intensity as described above.

### 4.2. Collection of Headspace Volatiles

Inoculated liquid cultures (100 mL) were transferred into 250 mL Erlenmeyer flasks and analyzed in dynamic headspace mode by OSSA. Thus, air was pumped through a cleaning charcoal filter over the stirred culture onto collection tubes at room temperature for one to four hours. Compounds in the gas phases of the cultures in the stationary phase were adsorbed on Tenax TA desorption tubes (Gerstel GmbH & Co.KG, Mülheim an der Ruhr, Germany) using a pump (MB-21E, Senior Flexonics Inc., Kassel, Germany). The analytes were desorbed by thermodesorption, trapped by cryofocusing and analyzed by GC/MS. Three biological replicates of each algal strain were performed for headspace sampling and GC/MS analyses. 

### 4.3. Experimental Procedures

#### 4.3.1. General Experimental Procedures

Chemicals were purchased from Sigma Aldrich (Taufkirchen, Germany), TCI (Eschborn, Germany) or from abcr GmbH (Germany) and used without further purification. The solvents were purified by distillation and dried according to usual standard laboratory methods. Reactions with air- and moisture-sensitive compounds were carried out in vacuum-heated flasks under a nitrogen atmosphere. Solutions at 0 °C were obtained with the aid of an ice-water bath. Thin-layer chromatography (TLC) was carried out on silica gel-coated films Polygram^®^ SIL G/UV254 (Macherey-Nagel, layer thickness 0.2 mm). In addition to UV detection (254 nm), common staining reagents such as molybdophosphoric acid or potassium permanganate were used as staining solutions. Flash column chromatography was carried out on silica gel 60 Å (grain size 35–70 μm) from Fisher Scientific. The NMR spectra were recorded with Avance II 300 (300 MHz for ^1^H, 75 MHz for ^13^C) and Avance III 400 (400 MHz for ^1^H, 100 MHz for ^13^C) spectrometers from Bruker at room temperature. Tetramethylsilane served as the internal standard. The chemical shifts are given in ppm relative to the standard. The coupling constants *J* are given in Hertz (Hz). Full synthetic details of the synthesized compounds are given in the Supporting Information.

#### 4.3.2. GC/MS Analyses

GC/MS analyses of synthetic samples were performed on an Agilent 8860 gas chromatograph coupled to an Agilent 5977B mass selective detector. The measurements were carried out in a pulsed split mode with the following temperature program: 50 °C (5 min isothermal) start temperature, 20 °C/min heating rate, 320 °C (5 min isothermal) final temperature. The analyses of the headspace extracts of the natural samples were performed on an Agilent 7890B gas chromatograph with a 5977A mass selective detector. The measurements were carried out with the following temperature program: 50 °C (5 min isothermal) start temperature, 5 °C/min heating rate, 320 °C (10 min isothermal) final temperature. Gas chromatographic separation was performed on fused-silica capillary columns HP-5MS (30 m × 0.25 mm ID × 0.25 μm film, Agilent Technologies, Santa Clara, CA, USA). Helium was used as carrier gas with a volume flow of 1.2 mL/min and ionization was carried out by electron impact ionization at 70 eV for both instruments. The GC/MS instrument for the headspace analysis was equipped with a thermal desorption unit (TDU 2, Gerstel GmbH & Co.KG, Mülheim an der Ruhr, Germany), a PTV inlet with a cooled injection system (CIS 4, Gerstel GmbH & Co.KG, Mülheim an der Ruhr, Germany) and a multipurpose sampler (MPS 2 XL, Gerstel GmbH & Co.KG, Mülheim an der Ruhr, Germany). The analytes were desorbed from Tenax TA desorption tubes under the following temperature program: initial temperature: 30 °C (delay time: 0.80 min, initial time: 0.10 min), 60 °C/min heating rate, 280 °C (5 min isothermal) final temperature. The analytes were cryofocused in the CIS under the following temperature program: initial temperature: −100 °C (equilibration time: 0.50 min, initial time: 0.01 min), 12 °C/s heating rate, 300 °C (3 min isothermal) final temperature. The analytes were desorbed in a splitless mode and the PTV inlet was in a solvent vent mode (vent flow: 40 mL/min, vent pressure: 7.70 psi until 0.01 min, purge flow to split vent: 50 mL/min at 0.76 min, 45 s splitless time). The TDU transfer temperature was set at 300 °C with a fixed transfer temperature mode. The TDU was cooled with a UPC Plus (Gerstel GmbH & Co.KG, Mülheim an der Ruhr, Germany) equipped with ethanol and the CIS was cooled with liquid nitrogen. Gas chromatographic retention indices were determined from a homologous series of n-alkanes (C_8_–C_40_). The *m*/*z* values are listed in unit masses and the relative intensities in %.

## 5. Conclusions

A sensitive headspace detection method allowed for the first time the analysis of VOCs released from xenic and axenic *P. cordatum* strains by GC/MS. The results revealed a large difference in the compound composition between the two strains with a low overlap of compounds. While in the axenic algae 52 compounds were detected, only 16 were found when the algae were cocultured with bacteria. The lactones **30**, **31** and **32** are new natural products, while compound **11**, the apocarotenoids **7**, **12**, **17**–**20** and **27** and the aromatic compounds **21**, **22**, **24** and **25** have not previously been reported as algal constituents. 

The bacterial presence largely influences the VOC composition, maybe by the uptake of the VOCs as DOM or direct interaction by the exchange of compounds and influence on the physiology of the algae. The resulting volatile bouquet can be potentially used as a signal indicating the algal physiological state. 

## Figures and Tables

**Table 1:** VOCs identified in the headspace extracts of xenic *Prorocentrum cordatum* CCMP 1529.

Compound	*I* (exp) ^a^	*I* (lit) ^b^	Identification ^c^	Rep 1	Rep 2	Rep 3
Benzoxazole (**10**)	1019	1067	ms, ri	x	x	x
2,2,6-Trimethylcyclohexan-1-one (**1**)	1035	1035	ms, ri		x	x
Unknown compound M 138 or M 154 (**a**)	1042			x	x	x
2,6,6-Trimethylcyclohex-2-en-1-one (**2**)	1060	1060	ms, ri, syn	x	x	x
3,5,5-Trimethylcyclohex-3-en-1-ol (**3**)	1064	1064	ms, ri, syn	x	x	x
α-Isophorone (**4**)	1120	1120	ms, ri	x	x	x
4-Oxoisophorone (**5**)	1144	1142	ms, ri	x	x	x
2-Hydroxyisophorone (**6**)	1148	1150	ms, ri		x	x
2-Hydroxy-4,4,6-trimethylcyclohexa-2,5-dienone (**7**)	1163	1165	ms, ri		x	x
2,3-Dimethyl-2-hepten-4-olide (**11**)	1318	1322	ms, ri, syn		x	x
Unknown compound M 152 or M 180 (**b**)	1327				x	x
Unknown compound M 166 (**c**)	1347			x	x	x
Unknown compound (**d**)	1368			x	x	x
Unknown compound M 210 (**e**)	1482			x	x	x
*trans*-β-Ionone (**8**)	1488	1486	ms, ri	x	x	x
Dihydroactinidiolide (**9**)	1533	1532	ms, ri		x	x

^a^ The gas chromatographic linear retention indices (*I*) listed are average values of the measurements of all replicates. ^b^ Literature values of *I* were obtained from the NIST Chemistry WebBook [36] or from our own database. ^c^ The compounds were identified based on comparison of the mass spectrum to a database spectrum (ms), comparison of *I* to a published value on the same or similar GC-fused silica capillary phase (ri) or comparison to a synthetic or commercially (syn) available reference compound. M 152 indicates the likely molecular ion in the mass spectra of unknown compounds. (**a**)–(**e**): unknown compounds. Rep = replicate; exp = experimental; lit = literature.

**Table 2:** VOCs identified in the headspace of axenic *Prorocentrum cordatum* CCMP 1329.

Compound	*I* (exp) ^a^	*I* (lit) ^b^	Identification ^c^	Rep 1	Rep 2	Rep 3
1-Octen-3-ol (**26**)	982	980	ms, ri	x	x	x
6-Methyl-5-hepten-2-ol (**27**)	995	994	ms, ri	x	x	x
Benzoxazole (**10**)	1019	1067	ms, ri	x	x	x
2,2,6-Trimethylcyclohexan-1-one (**1**)	1035	1035	ms, ri	x	x	
Benzyl alcohol (**23**)	1036	1036	ms, ri		x	x
Unknown compound M 150(**f**)	1045		ms, ri	x	x	x
2,6,6-Trimethylcyclohex-2-en-1-one (**2**)	1060	1060	ms, ri, syn	x	x	x
3,5,5-Trimethylcyclohex-3-en-1-ol (**3**)	1064	1065	ms, ri, syn	x	x	x
Methyl benzoate (**24**)	1095	1095	ms, ri	x	x	x
3,5-Dimethylcyclohex-2-en-1-one (**12**)	1101	1099	ms, ri, syn	x	x	x
2-Hydroxy-2,6,6-trimethylcyclohexan-1-one (**13**)	1108	1109	ms, ri, syn	x	x	x
α-Cyclocitral (**15**)	1117	1116	ms, ri	x	x	x
Unknown compound M 98 (**g**)	1122			x	x	x
(2*E*,6*Z*)-Nonadienal (**28**)	1154	1154	ms, ri, syn	x		x
Unknown compound M 147 (**h**)	1156			x	x	x
(*E*)-2-Nonenal (**29)**	1160	1160	ms, ri	x	x	x
α-Cyclogeraniol (**17**)	1175	1184	ms, ri	x	x	x
Benzyl(methyl)sulfane (**21**)	1181	1183	ms, ri		x	x
Unknown compound M 173 (**i**)	1198			x	x	x
Safranal (**14**)	1200	1201	ms, ri		x	x
β-Cyclogeraniol (**18**)	1207	1209	ms, ri, syn	x	x	x
Unknown compound (**j**)	1217			x	x	x
β-Cyclocitral (**16**)	1222	1222	ms, ri	x	x	x
Cyclohexyl isothiocyanate (**33**)	1233	1232	ms, ri, syn	x	x	x
Unknown compound M 175 (**k**)	1236			x	x	x
Unknown compound M 176 (**l**)	1262			x	x	x
Propyl benzoate (**25**)	1271	1272	ms, ri	x	x	x
Unknown compound M 161 (**m**)	1282			x	x	x
Unknown compound (**n**)	1293			x	x	x
Unknown compound M 166 (**o**)	1308			x	x	x
2,3-Dimethyl-2-hepten-4-olide (**11**)	1319	1322	ms, ri, syn	x	x	x
(6*E*,8*E*)-Megastigma-4,6,8-triene (**19**)	1342			x	x	x
Unknown compound M 166 (**c**)	1348			x	x	x
*trans-*3,7-Dimethyl-4-octanolide (**31**)	1356	1358	ms, ri, syn	x	x	x
(6*Z*,8*E*)-Megastigma-4,6,8-triene (**20**)	1363	1358		x	x	x
Unknown compound (**d**)	1368			x		x
*cis*-3,7-Dimethyl-4-octanolide (**32**)	1387	1389	ms, ri, syn	x	x	x
Unknown compound M 189 (**p**)	1391			x	x	x
3,7-Dimethyl-2-octen-4-olide (**30**)	1426	1429	ms, ri, syn	x	x	x
Unknown compound M 154 (**q**)	1427			x	x	x
Unknown compound M 204 (**r**)	1432			x	x	x
Unknown compound M 177 (**s**)	1478			x	x	x
Unknown compound M 210 (**e**)	1482				x	x
Unknown compound M 198 (**t**)	1501			x	x	x
Dihydroactinidiolide (**9**)	1533	1532	ms, ri	x	x	x
Unknown compound M 177 (**u**)	1542			x		x
Unknown compound M 253 (**v**)	1548			x	x	x
Unknown compound M 201 (**w1**)	1598			x	x	x
2-(Methylthio)benzo[*d*]thiazole (**22**)	1601	1589	ms, ri	x	x	x
Unknown compound M 205 (**x**)	1634			x		x
Unknown compound M 201 (**w2**)	1649			x	x	x
Unknown compound (**y**)	2056			x	x	x

^a^ The gas chromatographic linear retention indices (*I*) listed are average values of the measurements of all replicates. ^b^ Literature values of *I* were obtained from the NIST Chemistry WebBook [36] or from our own database. ^c^ The compounds were identified based on comparison of the mass spectrum to a database spectrum (ms), comparison of *I* to a published value on the same or similar GC-fused silica capillary phase (ri) or comparison to a synthetic or commercially (syn) available reference compound. M (150) indicates the likely molecular ion in the mass spectra of unknown compounds. (**c**)–(**y**): unknown compounds. **w1**, **w2** = isomers; Rep = replicate; exp = experimental; lit = literature.

## Data Availability

Mass spectra of synthesized target compounds **2**, **3**, **11**, **18**, **30**, **31** and **32** will be publicly available in the MACE mass spectral data repository after publication of this article [128,129].

## Supplementary Materials

The following supporting information can be downloaded at https://www.mdpi.com/article/10.3390/md20060371/s1, a pdf file containing detailed synthetic procedures, mass spectra and NMR spectra of synthesized compounds [37,38,39,40,41,42,43,44,127].
