# Supplementary material for: Identification of Volatiles of the Dinoflagellate Prorocentrum cordatum
Source: Mar Drugs. 2022 May 30;20(6):371. doi: 10.3390/md20060371 (PMC9230497; doi:10.3390/md20060371)
Supplement: Supplementary file 1 [file marinedrugs-20-00371-s001.zip › Supporting Information marinedrugs-1737025.pdf]

Supporting Information

Identification of Volatiles of the Dinoflagellate *Prorocentrum cordatum*

Diana Koteska[1], Selene Sanchez Garcia[2], Irene Wagner-Döbler[2], Stefan Schulz[1,*]

[1] Institute of Organic Chemistry, Technische Universität Braunschweig, 38106 Braunschweig, Germany

[2] Institute of Microbiology, Technische Universität Braunschweig, 38106 Braunschweig, Germany

Abstract: The dinoflagellate *Prorocentrum cordatum,* often called *P. minimum,* is a potentially toxic algae found in algal blooms. Volatile compounds released by the alga might carry important information, e. g. on its physiological state, and may act as chemical messengers. We report here the identification of volatile organic compounds emitted by two strains, xenic *P. cordatum* CCMP 1529 and axenic *P. cordatum* CCMP 1329. The volatiles released during cultivation were identified despite their low production rates using sensitive methods such as open-system-stripping analysis (OSSA) on Tenax TA desorption tubes, thermodesorption, cryofocussing and GC/MS-analysis. The analyses revealed 16 compounds released from the xenic strain and 52 compounds from the axenic strain. The majority of compounds were apocarotenoids, aromatic compounds and small oxylipins, but new natural products such as 3,7-dimethyl-4-octanolide were also identified and synthesized. The large difference between xenic and axenic algae in both amount and structure will be discussed.

Table of Contents

1. Synthetic procedures 2
2. Mass spectra 6
3. NMR Spectra 8
4. References 19

1. Synthetic procedures

β-Cyclogeraniol (18)

To a solution of β-cyclocitral (**16**) (200 mg, 1.31 mmol, 1.00 eq.) and $CeCl_3 \cdot 7\ H_2O$ (488 mg, 1.31 mmol, 1.00 eq.) in MeOH (3.6 mL) was slowly added $NaBH_4$ (49.6 mg, 1.31 mmol, 1.00 eq.) at 0 °C. The mixture was warmed to room temperature and stirred for 4 h. The reaction was quenched with H_2O, the aqueous layer was extracted three times with diethyl ether and the combined organic layers were dried over Na_2SO_4. The solvent was removed under reduced pressure to give the product **18** (196 mg, 1.27 mmol, 97%) as a clear, colourless oil [41].

I: 1209; ^{1}H-NMR (400 MHz, $CDCl_3$): δ = 4.14 (s, 2 H), 1.98 (t, *J* = 6.2 Hz, 2 H), 1.75 (s, 3 H), 1.63–1.55 (m, 2 H), 1.48–1.41 (m, 2 H), 1.04 (s, 6 H) ppm; ^{13}C-NMR (100 MHz, $CDCl_3$): δ = 137.7 (Cq), 133.6 (Cq) , 58.9 (CH_2), 39.4 (CH_2), 34.0 (Cq), 32.8 (CH_2), 28.5 (2 x CH_3), 19.6 (CH_3), 19.3 (CH_2) ppm; MS (EI, 70 eV): *m*/*z* (%) = 154 (43) $[M]^+$, 139 (32), 136 (19), 123 (82), 121 (100), 93 (79), 91 (23), 81 (30), 79 (48), 77 (16).

2-Hydroxy-2,6,6-trimethylcyclohexan-1-one (13)

A solution of *m*CPBA (77%, 587 mg, 2.62 mmol, 2.00 eq.) in $CHCl_3$ (7.0 mL) was slowly added to a solution of freshly distilled β-cyclocitral (**16**) (200 mg, 0.21 mL, 1.31 mmol, 1.00 eq.) in $CHCl_3$ (0.7 mL) at reflux under an atmosphere of nitrogen. The solution was stirred for 6 h at reflux, then cooled to room temperature and washed successively with aq. $NaHSO_3$ solution, 10% aq. NaOH solution and H_2O. The solvent was removed under reduced pressure and the resulting oil was hydrolysed with a 1% methanolic NaOH solution at room temperature for 2 h and then refluxed for 1.5 h. Methanol was evaporated under reduced pressure, the crude product was dissolved in $CHCl_3$, washed with brine and dried over $MgSO_4$. Purification by column chromatography on silica gel [pentane/diethyl ether (10:1)] gave the product **13** (162 mg, 1.04 mmol, 79%) as a clear, slightly yellow oil [37].

R_f: 0.25 (pentane/Et_2O 10:1); *I*: 1109; ^{1}H-NMR (300 MHz, $CDCl_3$): δ = 3.92 (br.s, 1 H), 2.14–2.05 (m, 1 H), 1.92–1.55 (m, 5 H), 1.41 (s, 3 H), 1.22 (s, 3 H), 1.15 (s, 3 H) ppm; ^{13}C-NMR (75 MHz, $CDCl_3$): δ = 218.8 (C=O), 75.9 (Cq), 44.3 (Cq), 40.7 (2 x CH_2), 27.3 (CH_3), 27.2 (CH_3), 25.7 (CH_3), 18.9 (CH_2) ppm; MS (EI, 70 eV): *m*/*z* (%) = 156 (3) $[M]^+$, 128 (42), 110 (42), 95 (61), 85 (22), 84 (18), 71 (100), 58 (35), 55 (15), 43 (40), 41 (15).

2,6,6-Trimethylcyclohex-2-en-1-one (2)

A solution of the hydroxyketone **13** (752 mg, 4.81 mmol, 1.00 eq.) in pyridine (7.0 mL) was added dropwise to a solution of MsCl (0.56 mL, 7.21 mmol, 1.50 eq.) in pyridine (15.0 mL) at 0 °C. The resulting reaction mixture was refluxed for 2 h, then cooled to room temperature and quenched with H_2O. The aqueous layer was extracted three times with diethyl ether, the combined organic layers were washed successively one time with H_2O, two times with a 20% $CuSO_4$-solution and one time with brine. The organic layer was dried over $MgSO_4$ and the solvent was removed under reduced pressure. Purification by column chromatography on silica gel [pentane/diethyl ether (40:1)] gave the product **3** (158 mg, 1.14 mmol, 24%) as a clear, colourless oil [38].

R_f: 0.24 (pentane/Et_2O 40:1); *I*: 1060; ^{1}H-NMR (300 MHz, $CDCl_3$): δ = 6.66–6.59 (m, 1 H), 2.37–2.28 (m, 2 H), 1.82 (dt, J = 0.5, 6.1 Hz, 2 H), 1.76 (dt, J = 1.4, 2.0 Hz, 3 H), 1.10 (s, 6 H) ppm; ^{13}C-NMR (75 MHz, $CDCl_3$): δ = 204.7 (C=O), 143.5 (CH), 133.7 (Cq), 41.2 (Cq), 36.6 (CH_2), 24.3 (2 x CH_3), 23.0 (CH_2), 16.5 (CH_3) ppm; MS (EI, 70 eV): *m*/*z* (%) = 138 (23) $[M]^+$, 110 (5), 95 (4), 83 (6), 82 (100), 55 (3), 54 (19), 53 (3), 41 (4), 39 (5).

7,9,9-Trimethyl-1,4-dioxaspiro[4.5]dec-7-ene (34)

As described by Babler et al. [39], a solution of α-isophorone (**4**) (2.76 g, 3.00 mL, 20.0 mmol, 1.00 eq.), glycol (3.72 g, 3.40 mL, 60.0 mmol, 3.00 eq.) and *p*-toluenesulfonic acid monohydrate (114 mg, 3.00 mol%) in toluene (50 mL) was refluxed for 7 h while removing water and glycol with a Dean-Stark trap and then stirred at room temperature for 15 h. The reaction was quenched with sat. $NaHCO_3$, the layers were separated and the aqueous layer was extracted three times with ethyl acetate. The combined organic layers were washed with brine, dried over $MgSO_4$ and the solvent was removed under reduced pressure. The crude product was purified by column chromatography on silica gel [pentane/EtOAc (80:1)] to give the product **34** (2.34 g, 12.8 mmol, 64%) as a clear, colourless oil.

R_f: 0.26 (pentane/EtOAc 80:1); ^{1}H-NMR (300 MHz, $CDCl_3$): δ = 5.19–5.14 (m, 1 H), 3.97–3.94 (m, 4 H), 2.16–2.12 (m, 2 H), 1.69–1.66 (m, 3 H), 1.62–1.59 (m, 2 H), 1.05 (s, 6 H) ppm; ^{13}C-NMR (75 MHz, $CDCl_3$): δ = 131.3 (CH), 128.0 (Cq), 109.2 (Cq), 64.1 (2 x CH_2), 43.6 (CH_2), 39.8 (CH_2), 34.2 (Cq), 30.4 (2 x CH_3), 23.4 (CH_3) ppm; MS (EI, 70 eV): *m*/*z* (%) = 182 (38) $[M]^+$, 167 (23), 96 (51), 95 (15), 87 (31), 86 (100), 81 (33), 79 (12), 67 (11), 41 (12).

3,5,5-Trimethylcyclohex-3-en-1-one (35)

The β-ketal **34** (498 mg, 2.73 mmol, 1.00 eq.) was dissolved in acetic acid and water (4:1; 13.7 mL) and stirred at room temperature for 4 h. The reaction mixture was then carefully poured into a cold $NaHCO_3$ solution (205 mL) and diluted with brine (137 mL). The aqueous layer was extracted three times with diethyl ether, the combined organic layers were dried over $MgSO_4$ and the solvent was removed under reduced pressure. A product mixture of **35** and α-isophorone (**4**) (356 mg, 2.58 mmol, 95%) was obtained as a clear, slightly yellow oil in the ratio of 5:1 as determined by NMR-analysis [39].

^{1}H-NMR (300 MHz, $CDCl_3$): δ = 5.46–5.39 (m, 1 H), 2.74–2.70 (m, 2 H), 2.32 (s, 2 H), 1.71 (dt, J = 1.5, 0.9 Hz, 3 H), 1.04 (s, 6 H) ppm; ^{13}C-NMR (75 MHz, $CDCl_3$): δ = 210.5 (C=O), 132.5 (CH), 129.0 (Cq), 53.2 (CH_2), 43.7 (CH_2), 36.4 (Cq), 29.6 (2 x CH_3), 22.6 (CH_3) ppm; MS (EI, 70 eV): *m*/*z* (%) = 138 (59) $[M]^+$, 123 (58), 96 (89), 95 (87), 81 (100), 79 (27), 67 (40), 53 (20), 41 (30), 39 (34).

3,5,5-Trimethylcyclohex-3-en-1-ol (3)

According to the published procedure of Rosini et al. [40], a solution of 3,5,5-trimethylcyclohex-3-en-1-one (**35**) (314 mg, 2.27 mmol, 1.00 eq.) in dry diethyl ether (1.0 mL) was slowly added to $LiAlH_4$ (57.3 mg, 1.51 mmol, 0.67 eq.) in dry diethyl ether (3.6 mL) under an atmosphere of nitrogen. The mixture was refluxed for 1 h, cooled to 0 °C and then water and 5% HCl solution were added successively. The organic layer was washed five times with brine, dried over $MgSO_4$ and the solvent was removed under reduced pressure. A product mixture of **3** and the isomer 3,5,5-trimethylcyclohex-2-en-1-ol (266 mg, 1.89 mmol, 84%) was obtained as a clear, colourless oil in the ratio of 5:1 as determined by NMR-analysis.

I: 1064; ^{1}H-NMR (300 MHz, $CDCl_3$): δ = 5.12–5.08 (m, 1 H), 3.99 (dddd, J = 11.6, 9.4, 5.6, 3.7 Hz, 1 H), 2.27–2.16 (m, 1 H), 1.92–1.66 (m, 3 H), 1.66–1.64 (m, 3 H), 1.54 (br. s, 1 H), 1.00 (s, 3 H), 0.98 (s, 3 H) ppm; ^{13}C-NMR (75 MHz, $CDCl_3$): δ = 131.7 (CH), 128.6 (Cq), 66.3 (CH), 46.1 (CH_2), 39.8 (CH_2), 34.1 (Cq), 31.4 (CH_3), 29.5 (CH_3), 23.3 (CH_3) ppm; MS (EI, 70 eV): m/z (%) = 140 (11) $[M]^+$, 125 (18), 122 (14), 107 (100), 96 (14), 91 (18), 81 (26), 79 (19), 55 (21), 41 (18), 39 (14).

5-Hydroxy-3,4-dimethylfuran-2(5*H*)-one (37)

Under an atmosphere of nitrogen, $LiAlH_4$ (331 mg, 8.72 mmol, 1.10 eq.) was suspended in dry THF (16 mL) and a solution of *t*-butanol (1.93 mg, 26.2 mmol, 3.30 eq.) in dry diethyl ether (8.0 mL) was added. This suspension was added dropwise over 30 min to a solution of 3,4-dimethylfuran-2,5-dione (**36**) (1.00 g, 7.93 mmol, 1.00 eq.) in dry diethyl ether (30 mL) at -10 °C and stirred for 1 h at this temperature. The solution was warmed to room temperature and stirred for 19 h, then cooled to 0 °C and quenched with 2 M H_2SO_4 (30 mL). The aqueous layer was extracted three times with diethyl ether and the combined organic layers were dried over Na_2SO_4. The crude product was purified by column chromatography on silica gel [pentane/EtOAc (5:1)] to give the product **37** (684 mg, 5.33 mmol, 67%) as a white solid [42].

R_f: 0.06 (pentane/EtOAc 5:1); ^{1}H-NMR (300 MHz, $CDCl_3$): δ = 5.91 (d, J = 4.6 Hz, 1 H), 5.21 (br. s, 1 H), 2.04–1.97 (m, 3 H), 1.84–1.78 (m, 3 H) ppm; ^{13}C-NMR (75 MHz, $CDCl_3$): δ = 173.5 (C=O), 156.5 (Cq), 125.8 (Cq), 98.7 (CH), 11.4 (CH_3), 8.3 (CH_3) ppm.

2,3-Dimethyl-2-hepten-4-olide (11)

Propylmagnesium bromide (**38**) (1 M in Me-THF, 1.87 mL, 1.87 mmol, 2.40 eq.) was added to a solution of 5-hydroxy-3,4-dimethylfuran-2(5*H*)-one (**37**) (100 mg, 0.78 mmol, 1.00 eq.) in dry and degassed THF (3.3 mL) under an atmosphere of nitrogen. The solution was stirred for 17 h at room temperature and quenched with 1 M HCl. The organic layer was extracted with diethyl ether three times and dried over $MgSO_4$. The solvent was removed under reduced pressure and the crude product was purified by column chromatography on silica gel [pentane/Et_2O (5:1)] to give the product **11** (104 mg, 0.67 mmol, 86%) as a clear, colourless oil [43].

R_f: 0.32 (pentane/ Et_2O 5:1); I: 1322; ^{1}H-NMR (300 MHz, $CDCl_3$): δ = 4.77–4.68 (m, 1 H), 1.94 (quin, J = 1.0 Hz, 3 H), 1.92–1.82 (m, 1 H), 1.82–1.79 (m, 3 H), 1.53–1.36 (m, 3 H), 0.95 (t, J = 7.2 Hz, 3 H) ppm; ^{13}C-NMR (75 MHz, $CDCl_3$): δ = 174.7 (C=O), 159.2 (Cq), 123.2 (Cq), 83.0 (CH), 34.2 (CH_2), 17.9 (CH_2), 13.8 (CH_3). 11.9 (CH_3), 8.4 (CH_3) ppm; MS (EI, 70 eV): m/z (%) = 155 (5), 154 (42) $[M]^+$, 125 (8), 112 (18), 111 (69), 84 (7), 83 (100); 43 (5), 55 (42), 39 (5).

3,7-Dimethyl-2-octen-4-olide (30)

1-Bromo-3-methylbutane (1.40 mL, 11.4 mmol, 2.60 eq.) in THF (20.0 mL) was added slowly to an I_2-activated suspension of magnesium (277 mg, 11.4 mmol, 2.60 eq.) in dry and degassed THF (0.6 mL) to generate the Grignard reagent **40**. The solution was refluxed for 1 h, then 5-hydroxy-4-methylfuran-2(5*H*)-one (**39**) (500 mg, 4.38 mmol, 1.00 eq.) in dry and degassed THF (19.0 mL) was slowly added and the reaction mixture was stirred for 3 h at reflux. The reaction mixture was cooled to room temperature and quenched by the addition of 1 M HCl. The aqueous layer was extracted three times with diethyl ether, the combined organic

layers were dried over $MgSO_4$ and the solvent was removed under reduced pressure. The crude product was purified by column chromatography on silica gel [pentane/diethyl ether (2:1)] to give the product **30** (627 mg, 3.73 mmol, 85%) as a clear, colourless oil.

R_f: 0.16 (pentane/ Et_2O 2:1); *I*: 1429; ^{1}H-NMR (300 MHz, $CDCl_3$): δ = 5.80 (quin, *J* = 1.5 Hz, 1 H), 4.88–4.78 (m, 1 H),2.06 (dd, *J* = 0.8, 1.5 Hz, 3 H), 1.99–1.86 (m, 1 H), 1.65–1.44 (m, 2 H), 1.37–1.15 (m, 2 H), 0.90 (d, *J* = 6.6 Hz, 3 H), 0.89 (d, *J* = 6.6 Hz, 3 H) ppm; ^{13}C-NMR (75 MHz, $CDCl_3$): δ = 173.4 (C=O), 168.6 (Cq), 116.9 (Cq), 84.8 (CH), 33.0 (CH_2), 29.7 (CH_2), 27.8 (CH), 22.5 (CH_3), 22.2 (CH_3), 13.9 (CH_3) ppm; MS (EI, 70 eV): *m*/*z* (%) = 168 (8) $[M]^+$, 112 (22), 99 (53), 98 (99), 97 (91), 81 (19), 69 (100), 43 (78), 39 (59), 55 (24).

***trans*-3,7-Dimethyl-4-octanolide (31) and *cis*-3,7-dimethyl-4-octanolide (32)**

To a solution of the lactone **30** (300 mg, 1.78 mmol, 1.00 eq.) and 10% Pd/C (30.0 mg, 10 wt.%) in EtOAc (4.0 mL) was introduced H_2 at 1 bar and the mixture was stirred at room temperature for 44 h. The mixture was filtered over silica gel and washed with diethyl ether. The solvent was removed under reduced pressure and the crude product was purified by column chromatography on silica gel [pentane/EtOAc (1:1)]. The products **31** (61.7 mg, 0.36 mmol, 20%) and **32** (205 mg, 1.21 mmol, 68%) were obtained as clear, colourless liquids [44]. The assignment of the *syn*- and *anti*-diastereomers was performed by comparison of the NMR data with published values [127].

Lactone **31**: R_f: 0.23 (pentane/ EtOAc 10:1); *I*: 1358; ^{1}H-NMR (400 MHz, $CDCl_3$): δ = 3.99 (ddd, *J* = 4.0, 7.3, 8.3 Hz, 1 H), 2.74–2.61 (m, 1 H), 2.27–2.17 (m, 2 H), 1.75–1.64 (m, 1 H), 1.48–1.37 (m, 1 H), 1.33–1.22 (m, 1 H), 1.14 (d, *J* = 6.5 Hz, 3 H), 0.91 (d, *J* = 6.6 Hz, 3 H), 0.90 (d, *J* = 6.6 Hz, 3 H) ppm; ^{13}C-NMR (100 MHz, $CDCl_3$): δ = 176.5 (C=O), 87.7 (CH), 37.1 (CH_2), 36.0 (CH), 34.6 (CH_2), 31.8 (CH_2), 27.9 (CH), 22.5 (CH_3), 22.3 (CH_3), 17.5 (CH_3) ppm; MS (EI, 70 eV): *m*/*z* (%) = 170 (5) $[M]^+$, 115 (11), 114 (13), 101 (11), 100 (11), 99 (100), 83 (19), 71 (28), 56 (11), 43 (16), 41 (11).

Lactone **32**: R_f: 0.18 (pentane/ EtOAc 10:1); *I*: 1389; ^{1}H-NMR (400 MHz, $CDCl_3$): δ = 4.40 (td, *J* = 5.3, 8.9 Hz, 1 H), 2.70 (dd, *J* = 7.8, 16.9 Hz, 1 H), 2.63–2.52 (m, 1 H), 2.23–2.17 (m, 1 H), 1.71–1.61 (m, 1 H), 1.61–1.57 (m, 1 H), 1.57–1.48 (m, 1 H), 1.47–1.34 (m, 1 H), 1.28–1.13 (m, 1 H), 1.02 (d, *J* = 7.0 Hz, 3 H), 0.91 (d, *J* = 6.6 Hz, 3 H), 0.91 (d, *J* = 6.6 Hz, 3 H) ppm; ^{13}C-NMR (100 MHz, $CDCl_3$): δ = 176.9 (C=O), 83.9 (CH), 37.6 (CH_2), 34.8 (CH_2), 33.0 (CH), 27.9 (CH), 27.7 (CH_2), 22.4 (2 x CH_3), 13.8 (CH_3) ppm; MS (EI, 70 eV): *m*/*z* (%) = 170 (3) $[M]^+$, 101 (10), 99 (100), 83 (22), 71 (23), 70 (11), 56 (14), 55 (13), 43 (18), 42 (14), 41(13).

2. Mass spectra

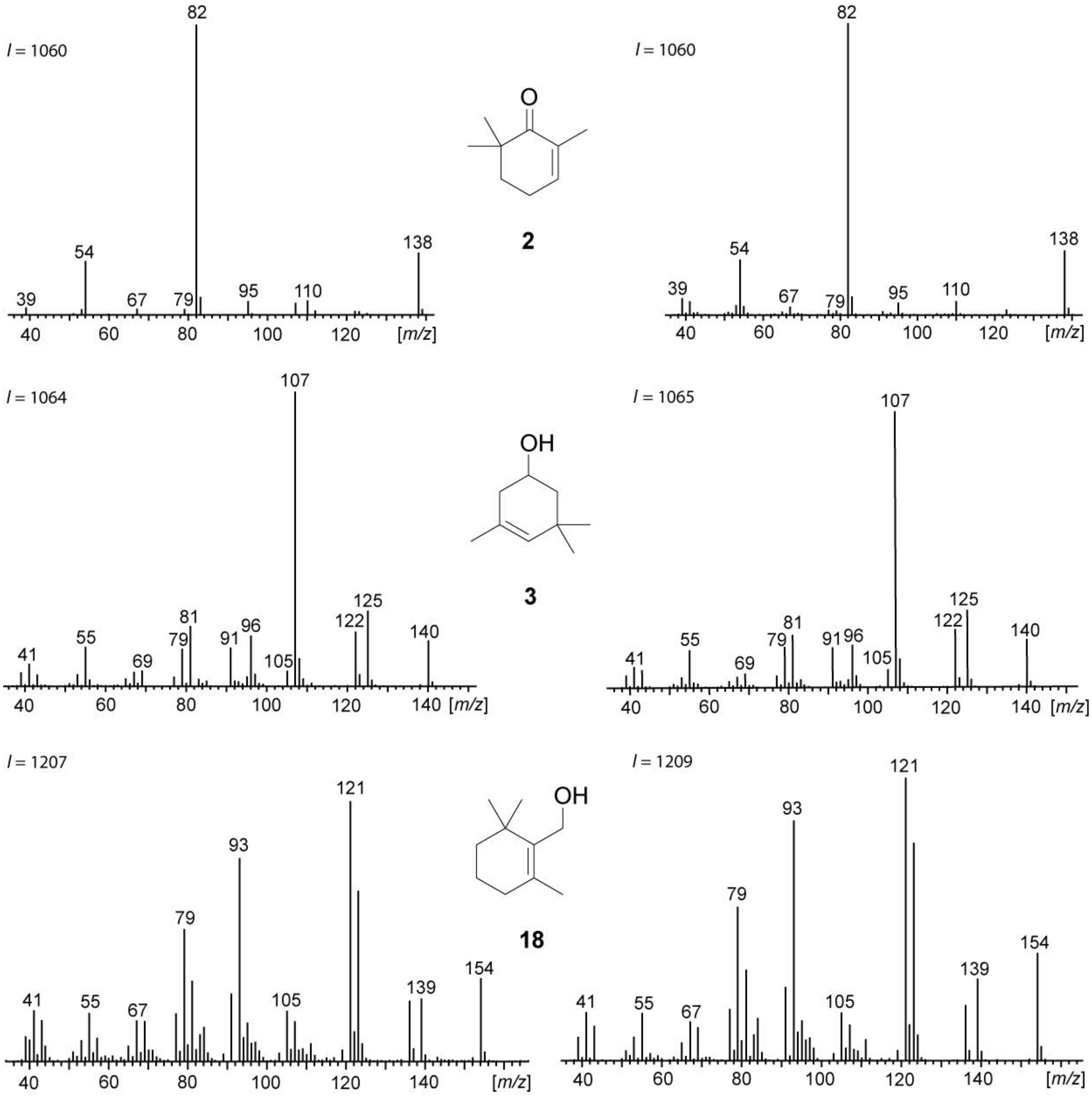

Figure S1: Comparison of the mass spectra and retention indices of the natural (left) and the synthetic compounds (right).

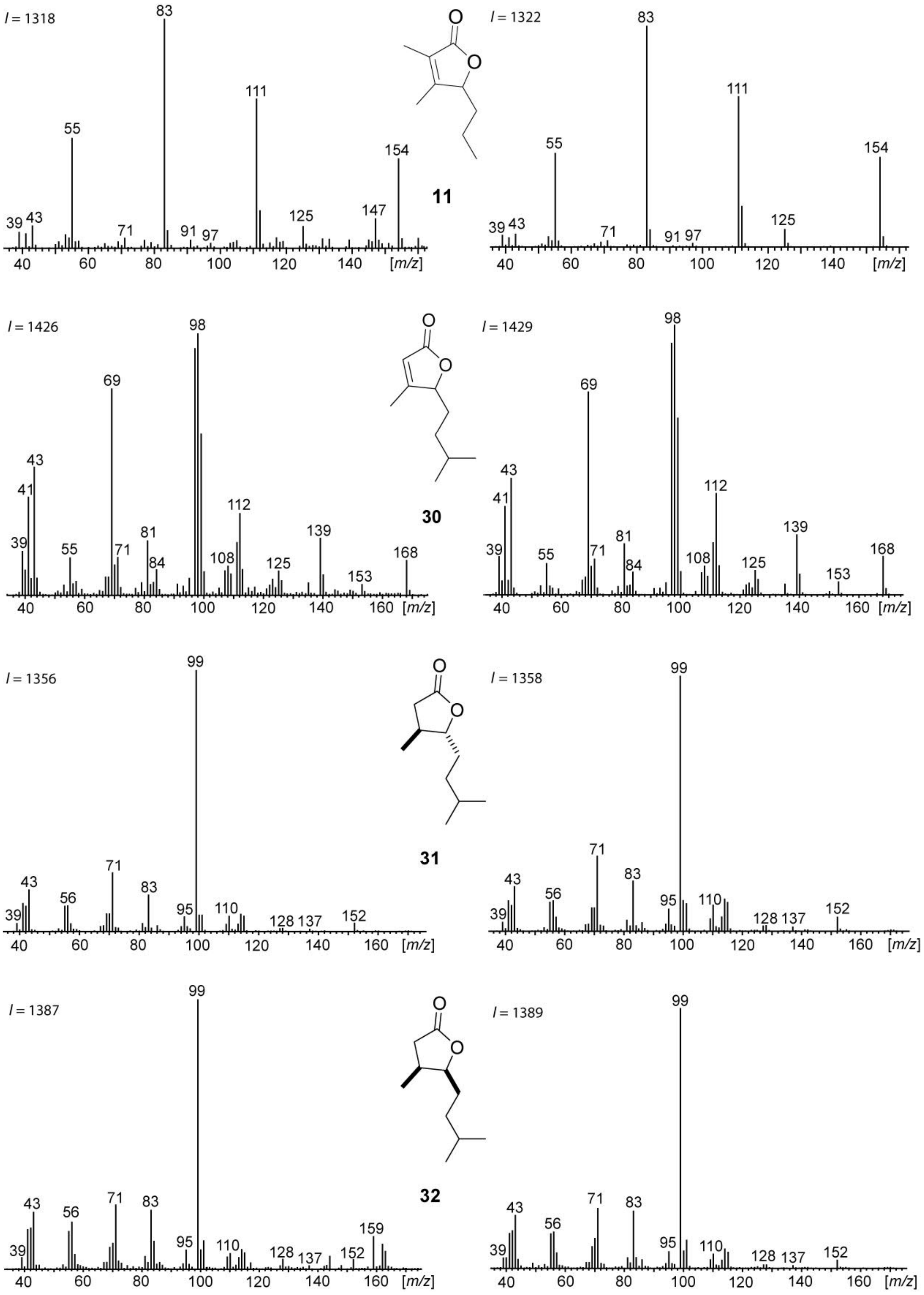

Figure S2: Comparison of the mass spectra and retention indices of the natural (left) and the synthetic compounds (right).

3. NMR Spectra

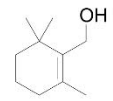

18

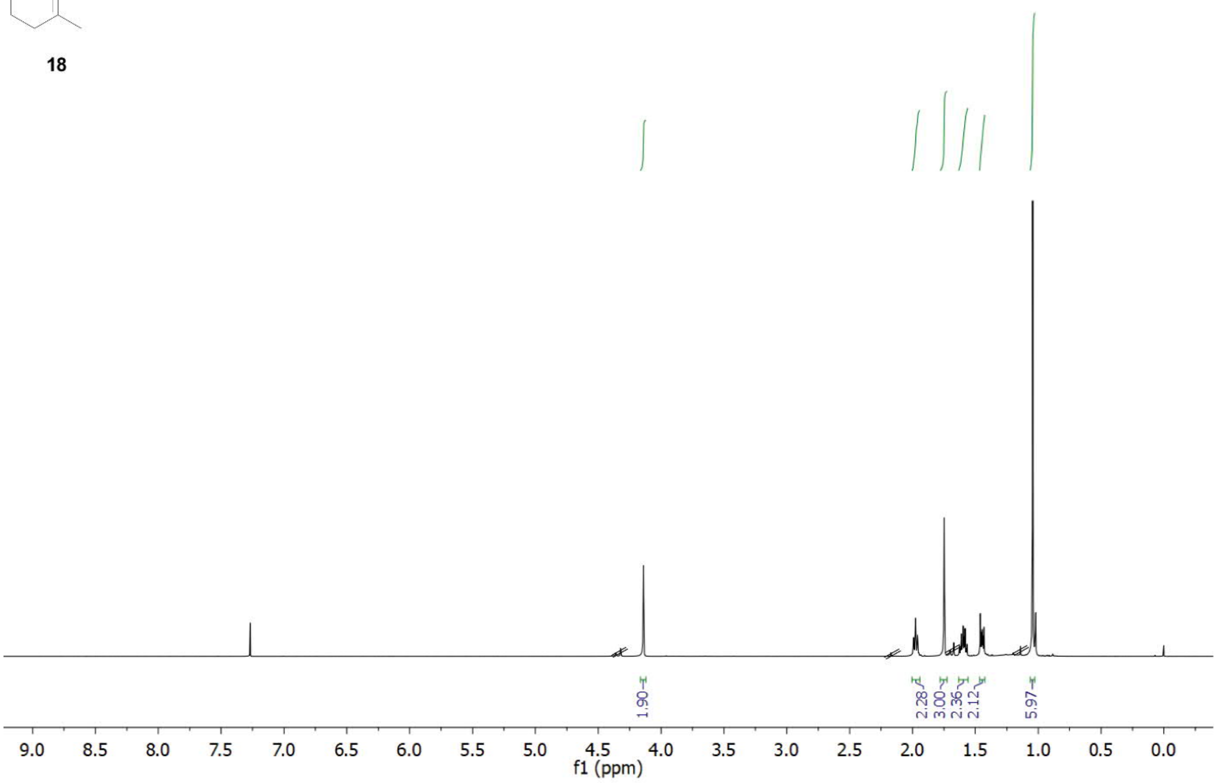

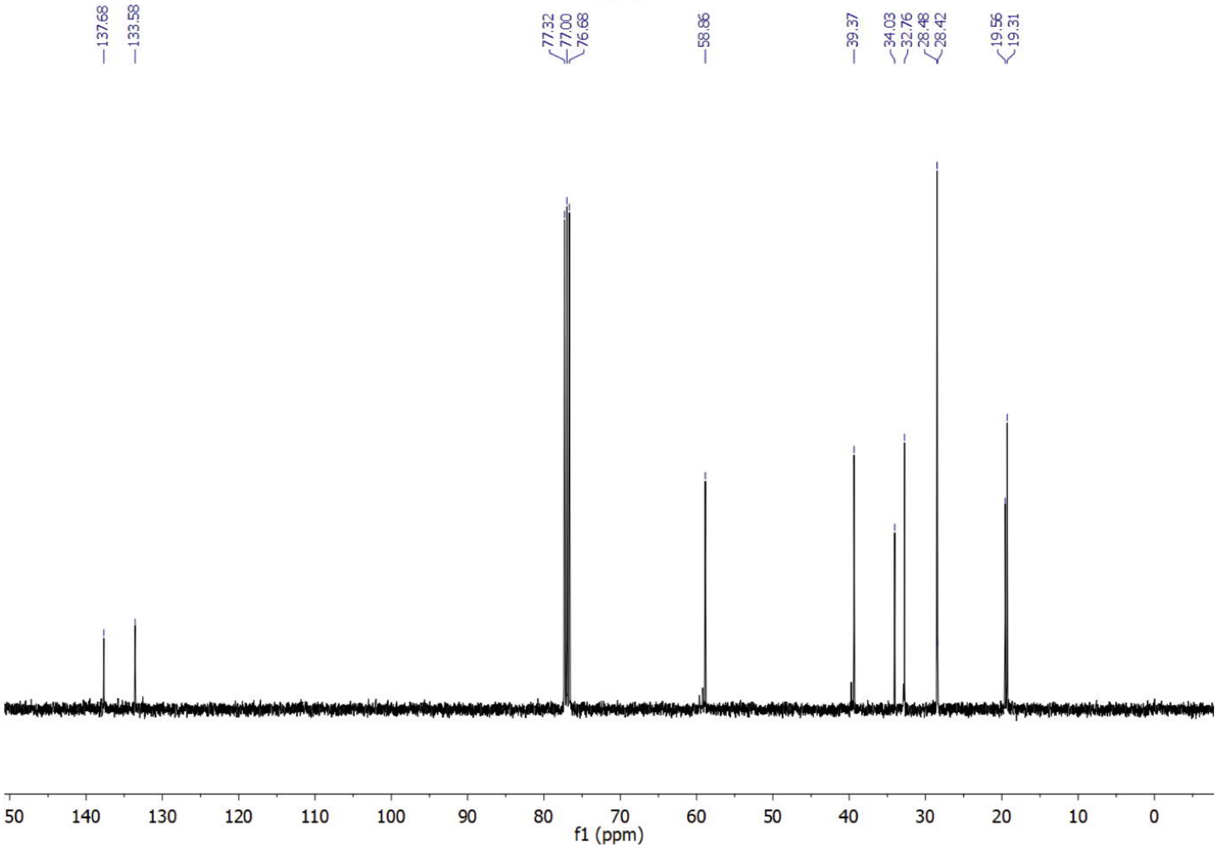

Figure S3. ^{1}H- and ^{13}C-NMR of β-cyclogeraniol (**18**).

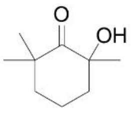

13

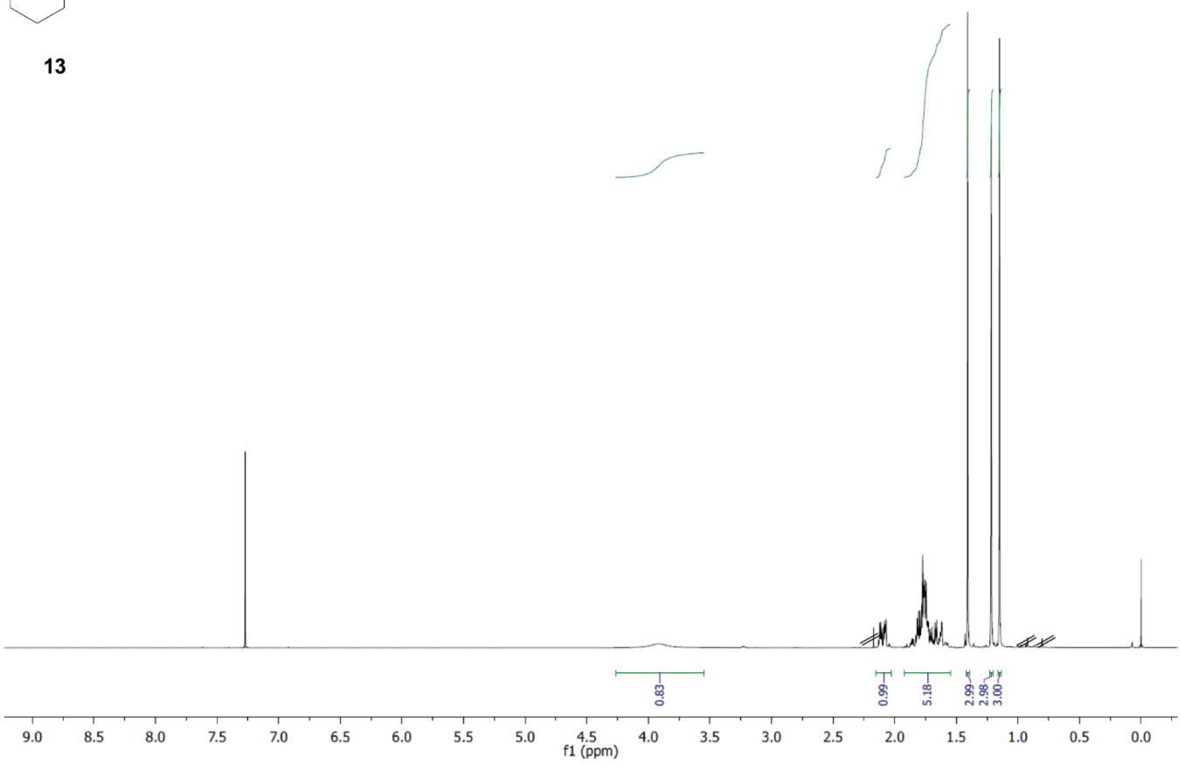

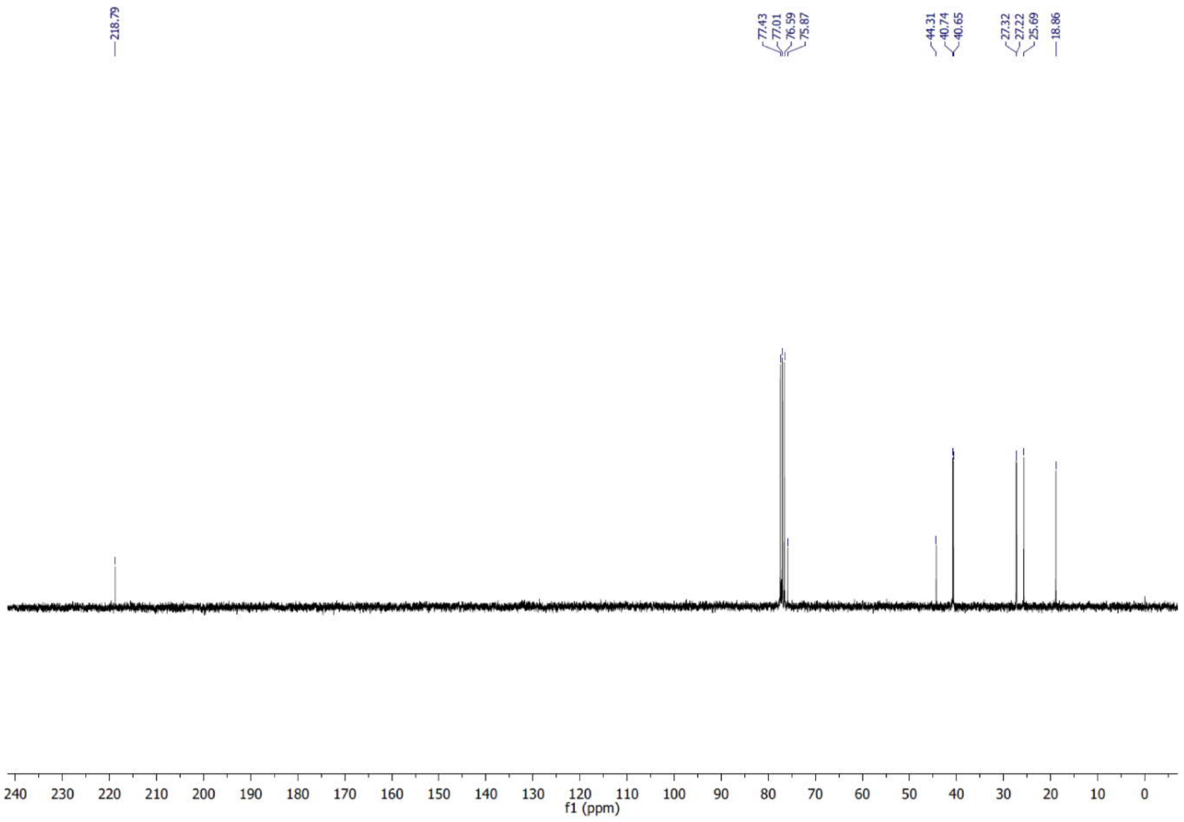

Figure S4. ^{1}H- and ^{13}C-NMR of 2-hydroxy-2,6,6-trimethylcyclohexan-1-one (**13**).

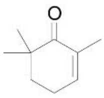

2

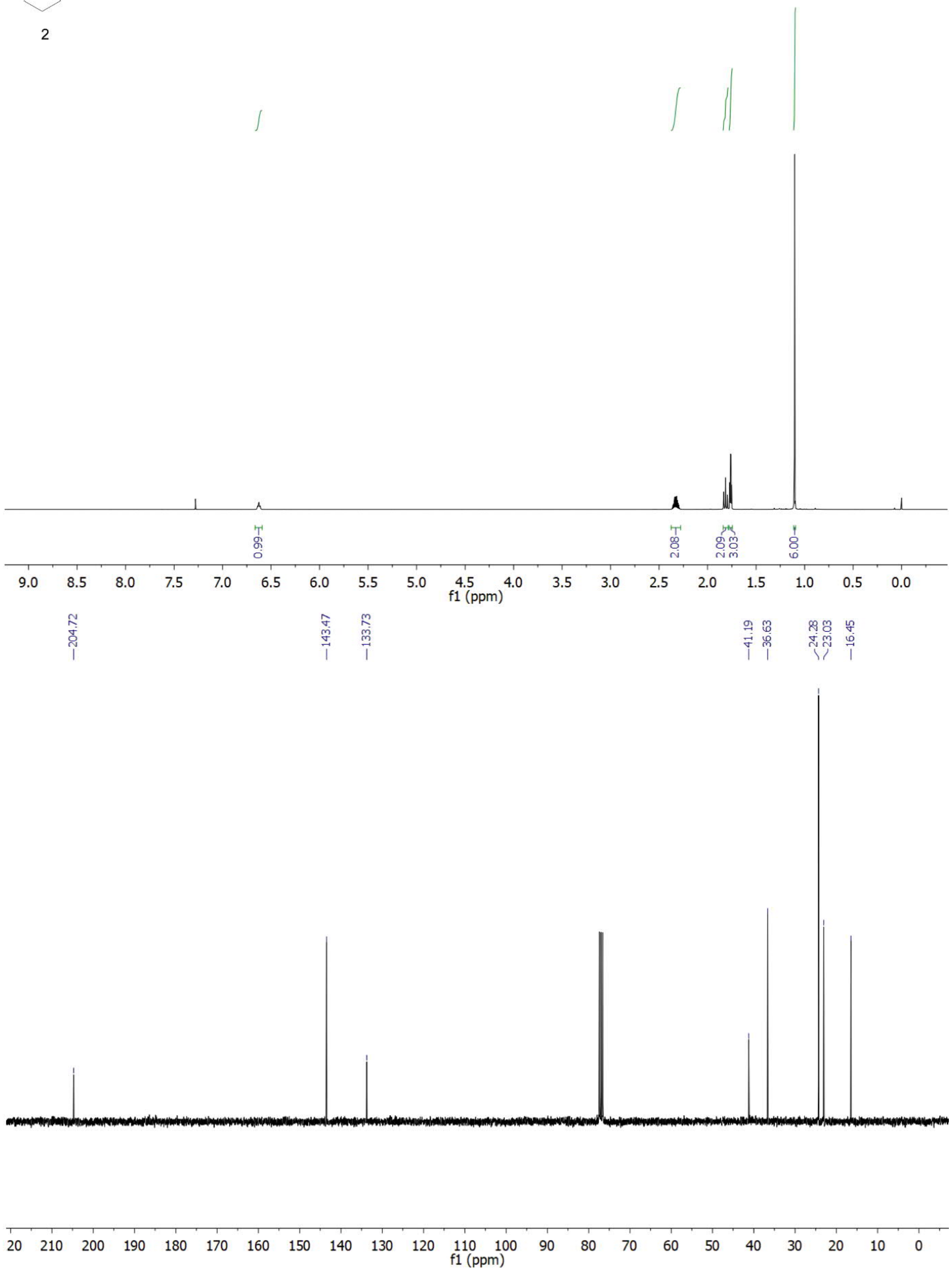

Figure S5. ^{1}H- and ^{13}C-NMR of 2,6,6-trimethylcyclohex-2-en-1-one (**2**).

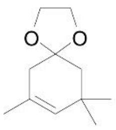

34

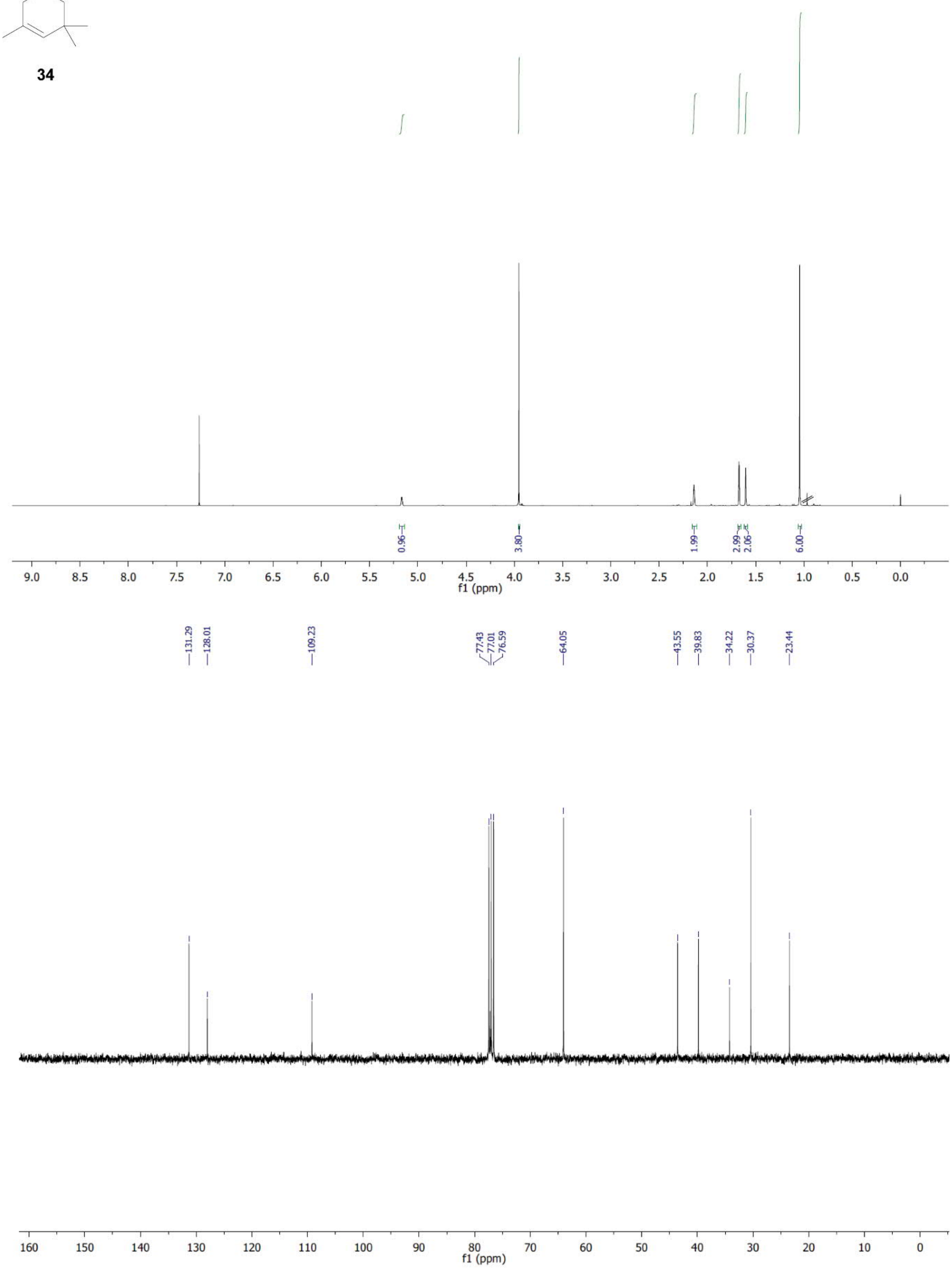

Figure S6. ^{1}H- and ^{13}C-NMR of 7,9,9-trimethyl-1,4-dioxaspiro[4.5]dec-7-ene (**34**).

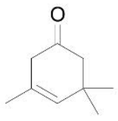

35

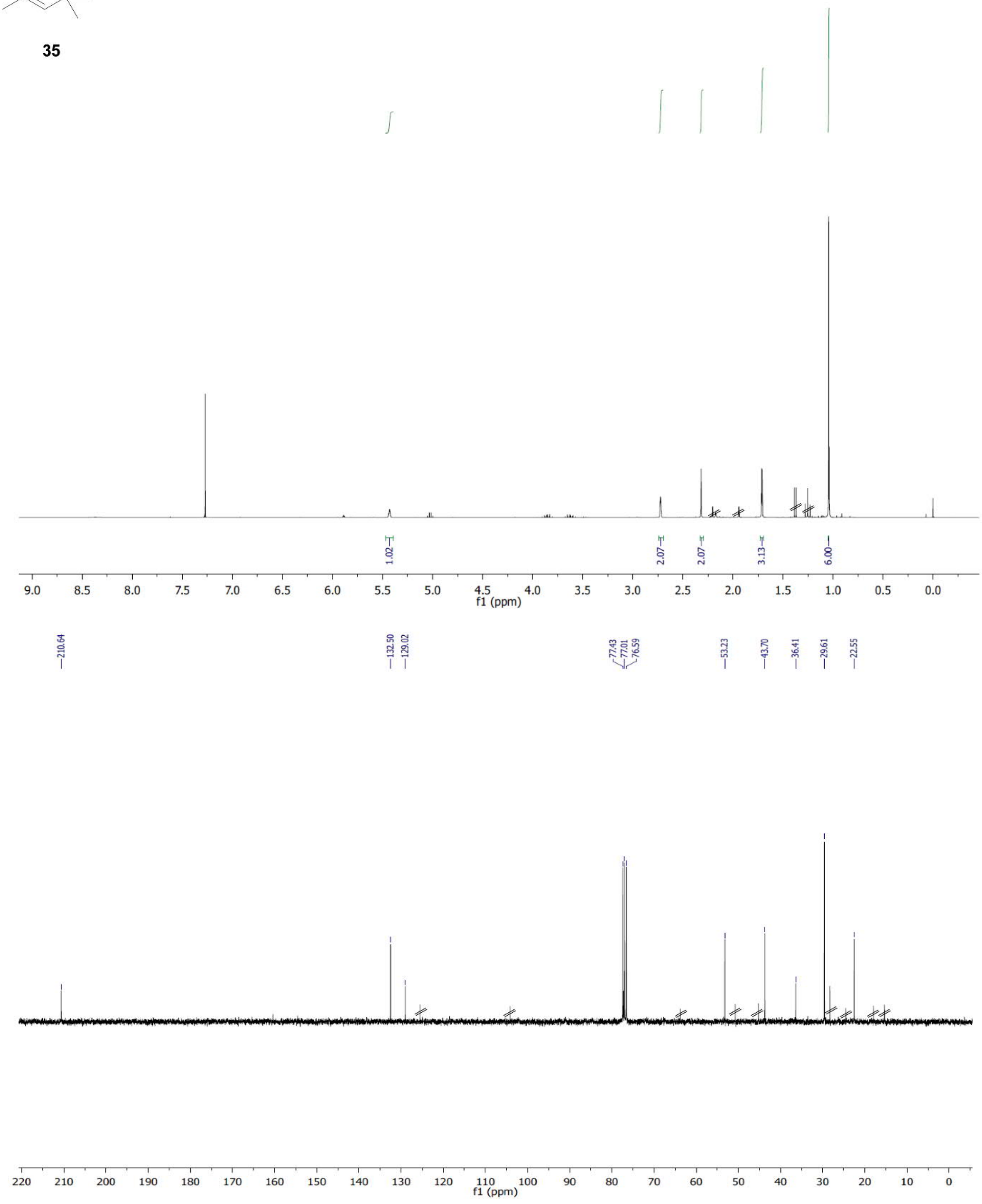

Figure S7. ^{1}H- and ^{13}C-NMR of 3,5,5-trimethylcyclohex-3-en-1-one (**35**).

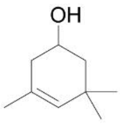

3

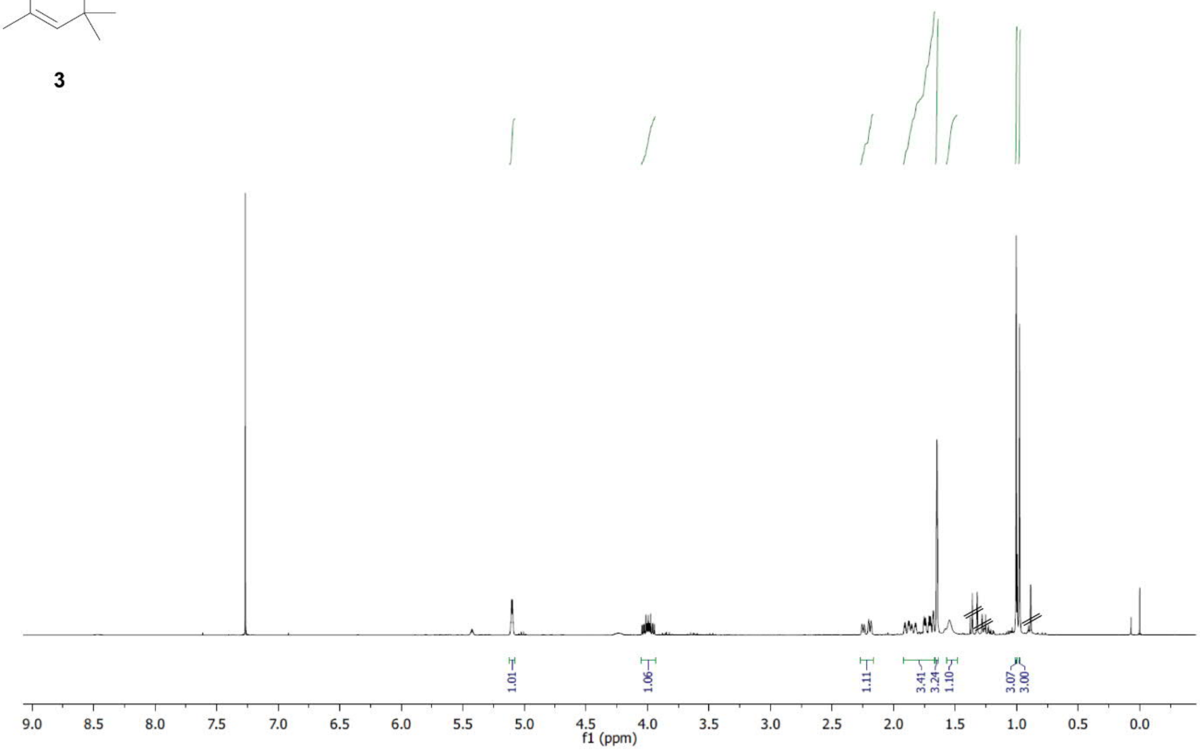

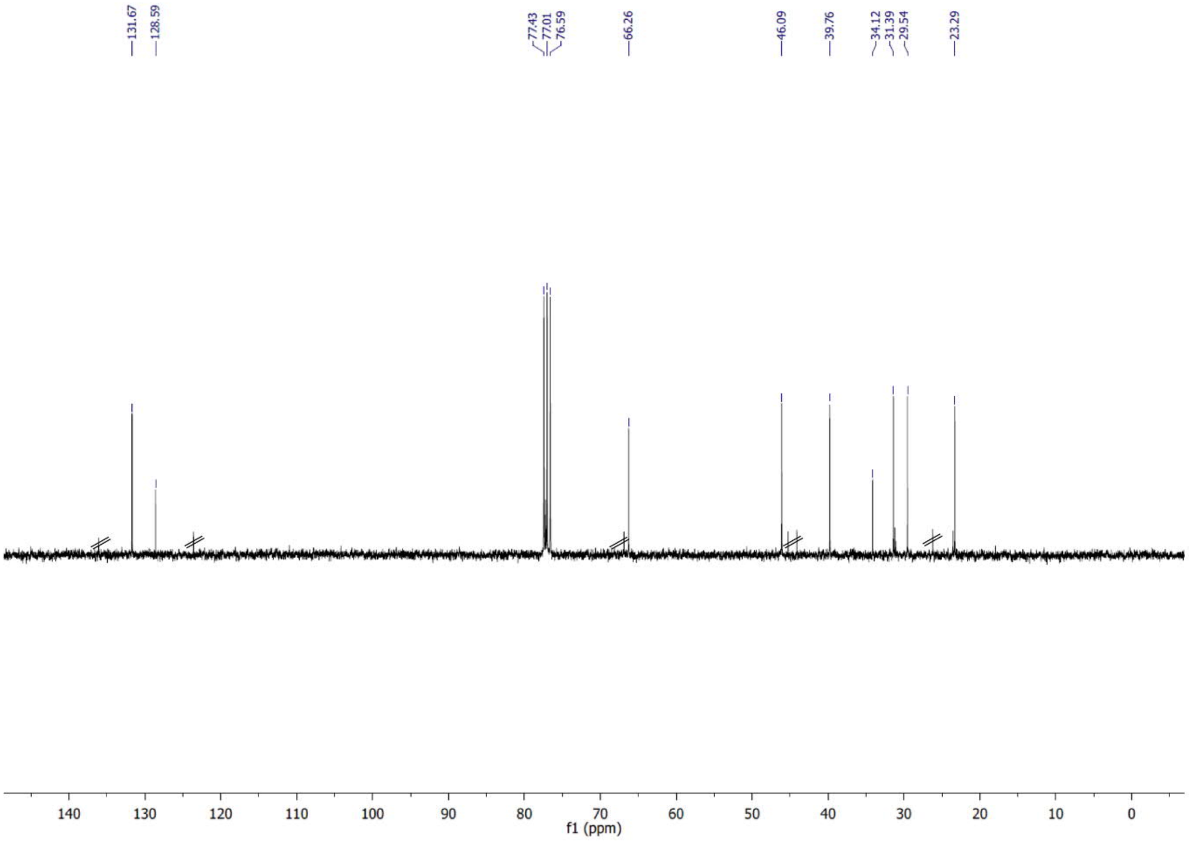

Figure S8. ^{1}H- and ^{13}C-NMR of 3,5,5-trimethylcyclohex-3-en-1-ol (**3**).

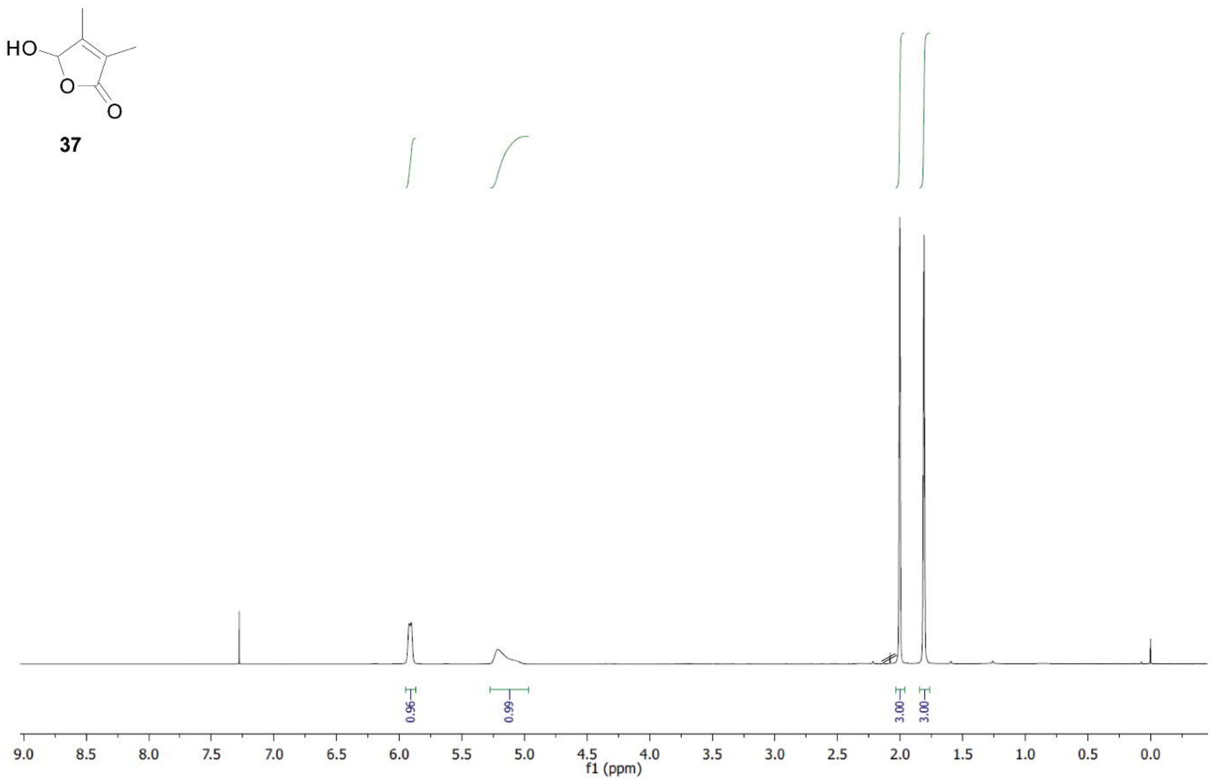

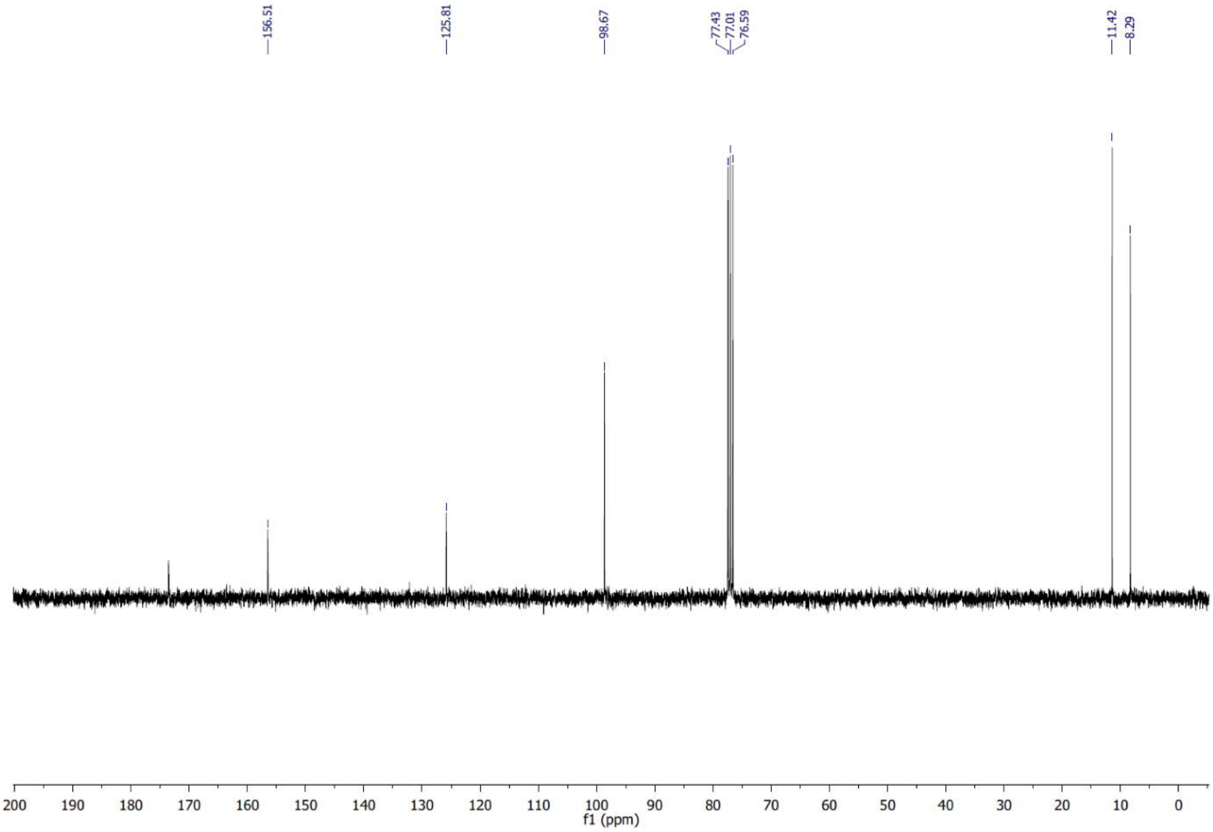

Figure S9. ^{1}H- and ^{13}C-NMR of 5-hydroxy-3,4-dimethylfuran-2(5*H*)-one (**37**).

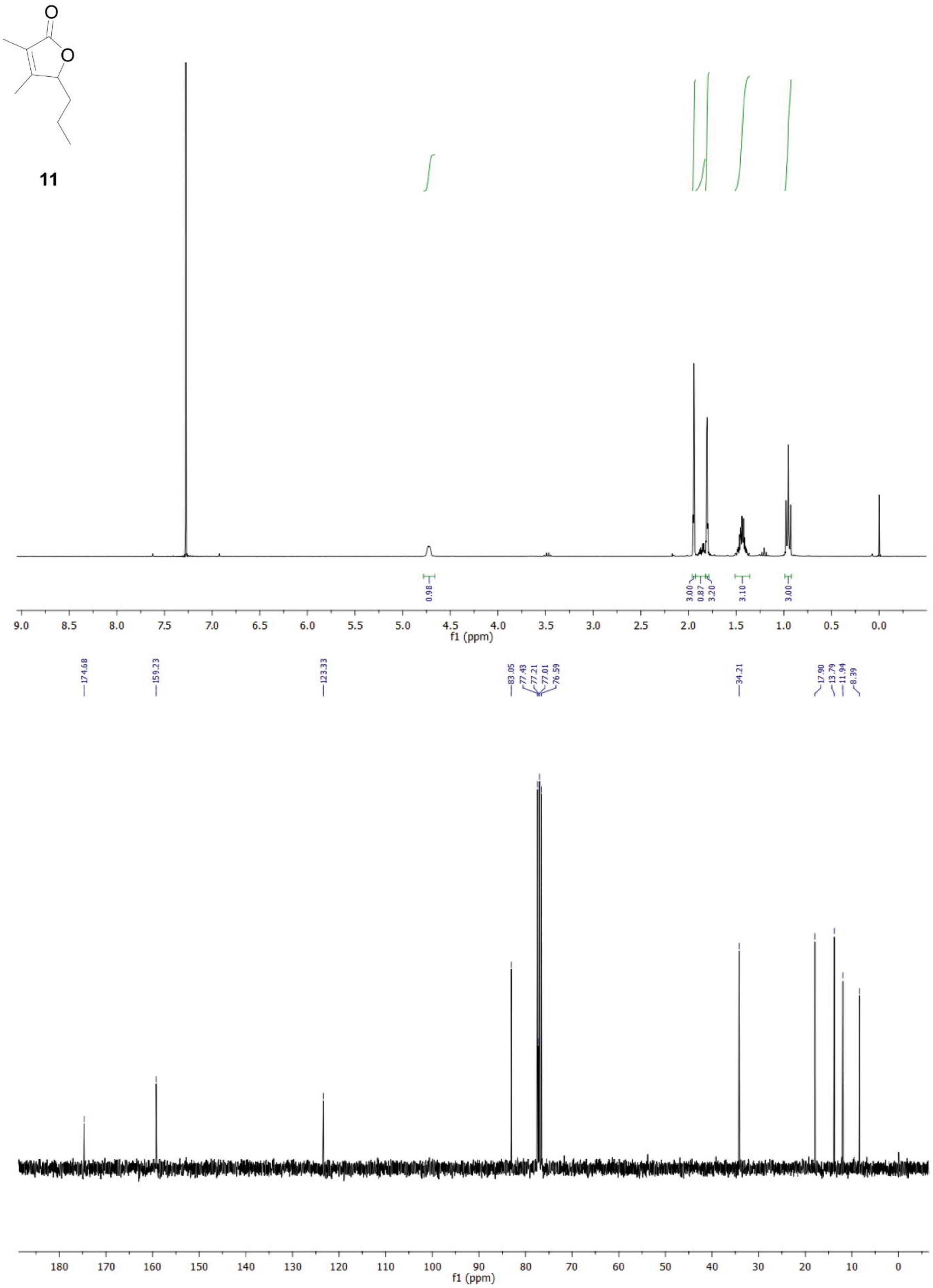

Figure S10. ^{1}H- and ^{13}C-NMR of 2,3-dimethyl-2-hepten-4-olide (**11**).

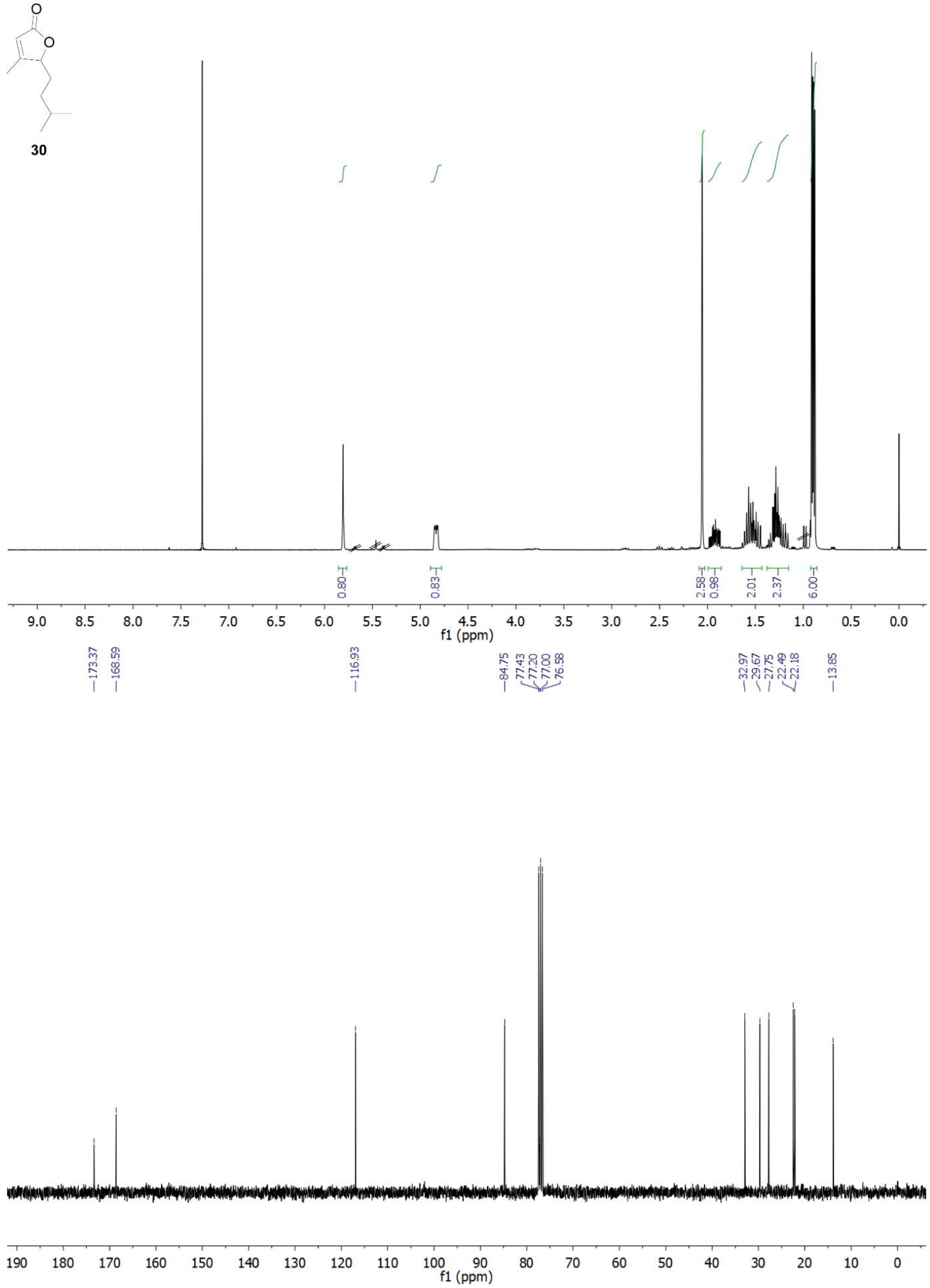

Figure S11. ^{1}H- and ^{13}C-NMR of 3,7-dimethyl-2-octen-4-olide (**30**).

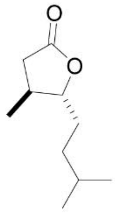

31

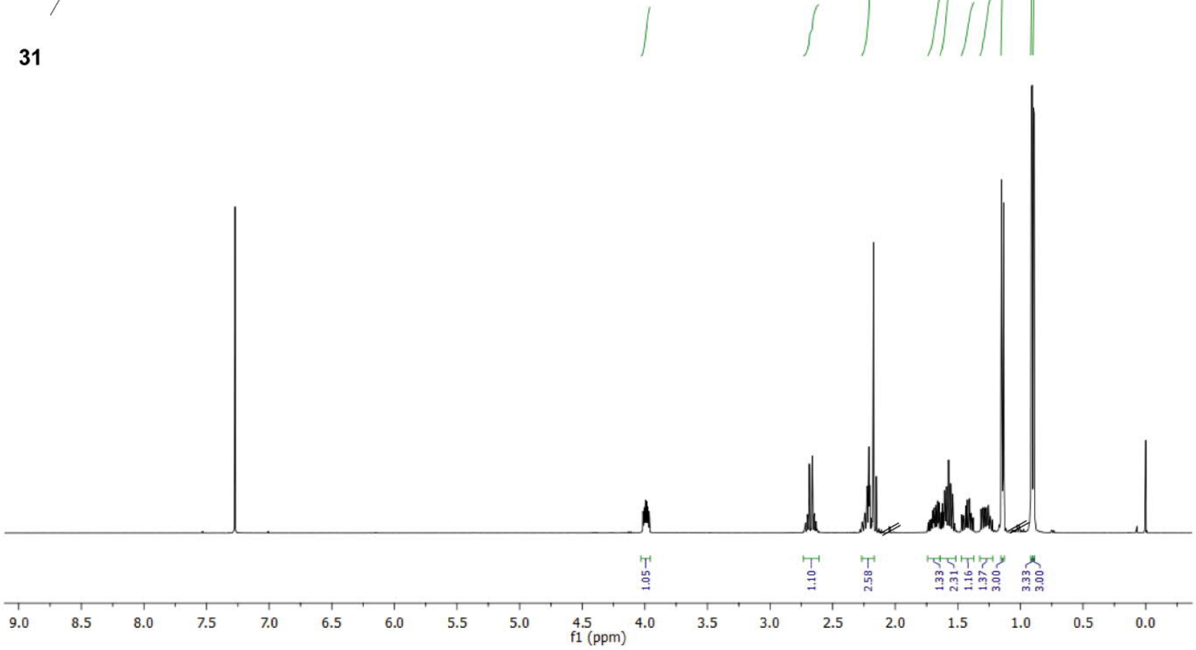

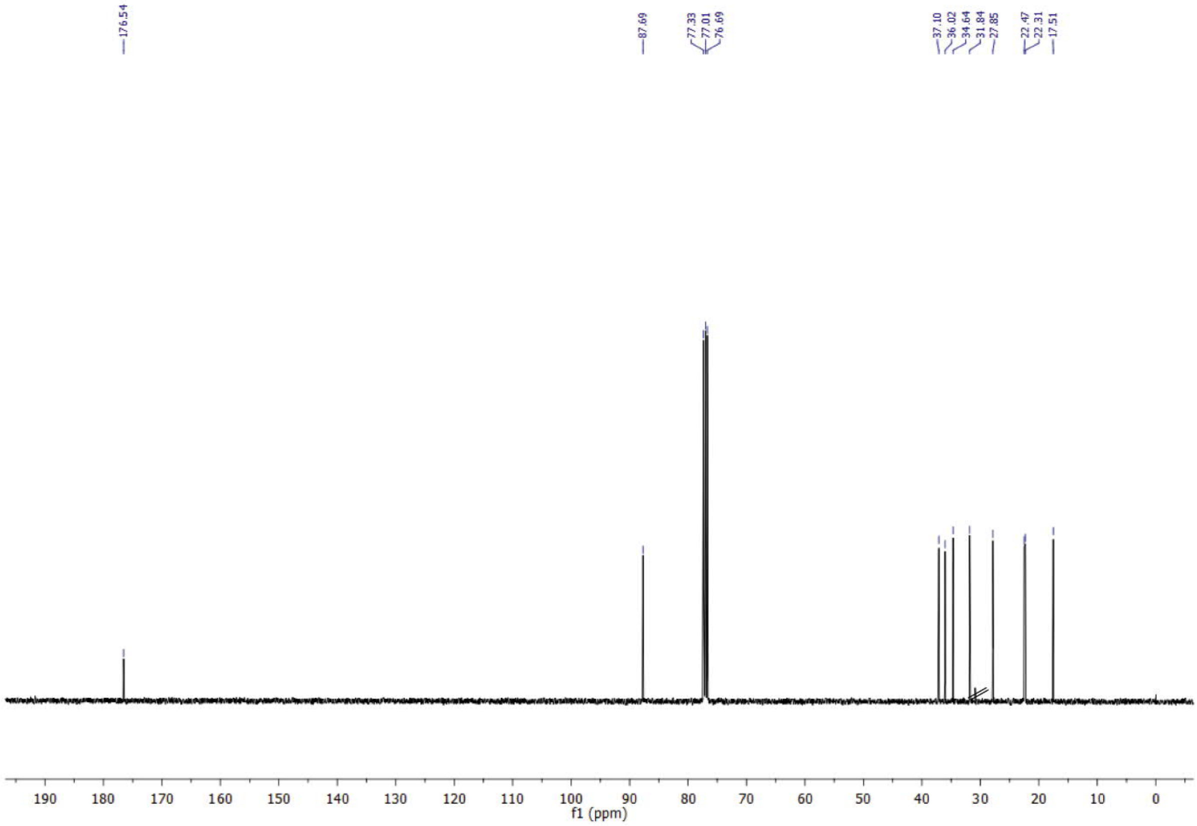

Figure S12. ^{1}H- and ^{13}C-NMR of *trans*-3,7-dimethyl-4-octanolide (**31**).

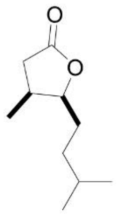

32

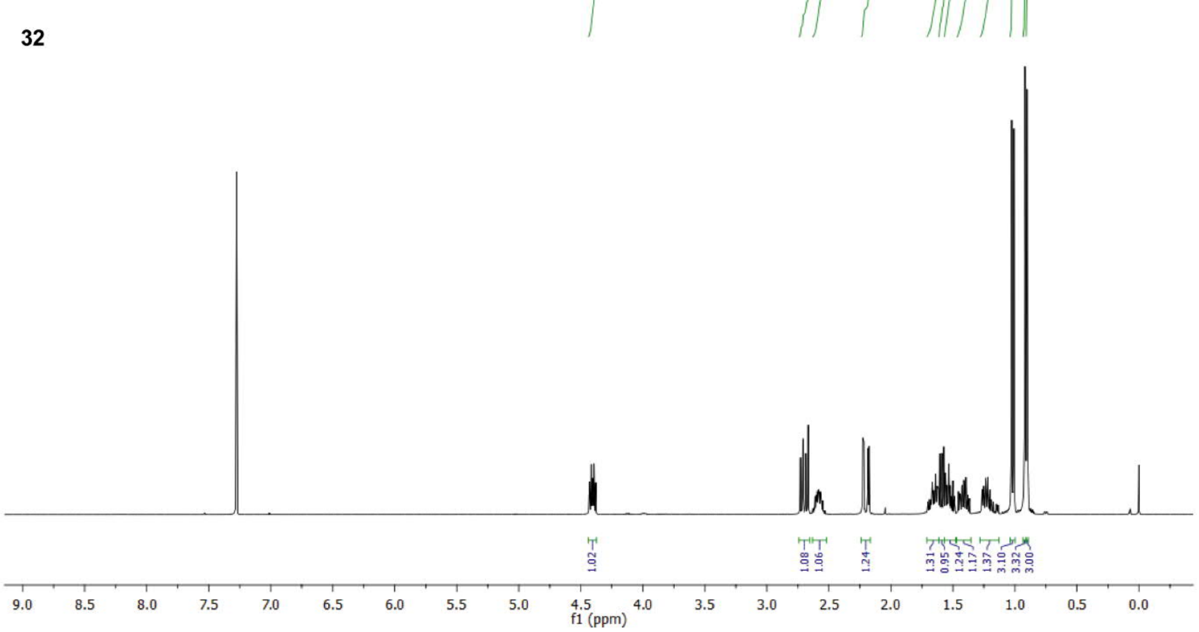

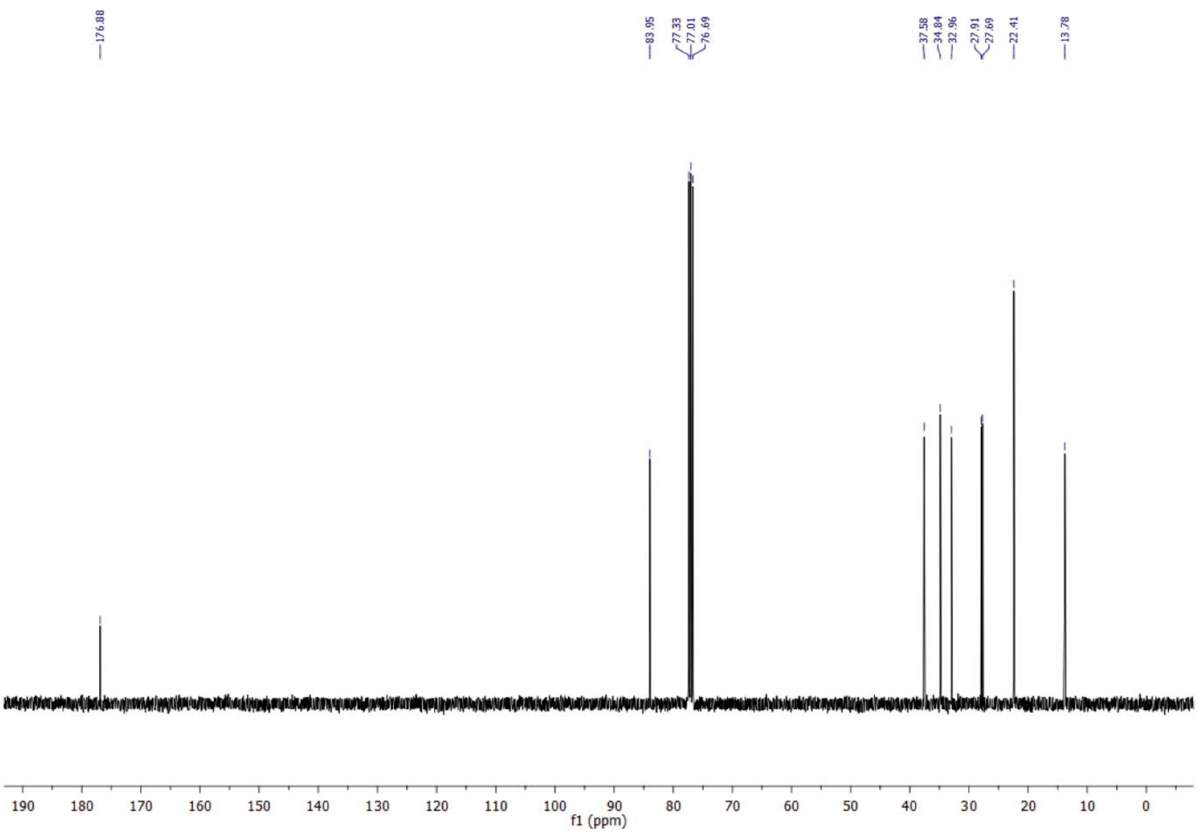

Figure S13. ^{1}H- and ^{13}C-NMR of *cis*-3,7-dimethyl-4-octanolide (**32**).

4. References

37. Subbaraju, G.V.; Manhas, M.S.; Bose, A.K. A Convenient Synthesis of 2-Hydroxy-2,6,6-trimethylcyclohexanone: A Versatile Intermediate. *Synthesis* **1992**, 816–818, doi:10.1055/s-1992-26231.
38. Constantino, M.G.; Donate, P.M.; Petragnani, N. An efficient synthesis of (+-)-abscisic acid. *J. Org. Chem.* **1986**, *51*, 253–254, doi:10.1021/jo00352a027.
39. Babler, J.H.; Malek, N.C.; Coghlan, M.J. Selective hydrolysis of α,β- and β,γ-unsaturated ketals: a method for deconjugation of β,β-disubstituted α,β-unsaturated ketones. *J. Org. Chem.* **1978**, *43*, 1821–1823.
40. Rosini, G.; Ballini, R.; Zanotti, V. Cycloaddition of dichloroketene with functionalized cycloalkenes, synthesis of bicyclo[4.2.0]octanone-3-yl derivatives and of 3 4-dicarbomethoxy-1-methylbicyclo [4.2.0]octan-7-one. *Tetrahedron* **1983**, *39*, 1085–1090.
41. Tomas, M.C. Aspects of thionitrites and nitric oxide in chemistry and biology. PhD thesis; University of London, London, 1999.
42. Schobert, R.; Barnickel, B. A Regioselective Tsuji-Trost Pentadienylation of 3-Allyltetronic Acid. *Synthesis* **2009**, *2009*, 2778–2784, doi:10.1055/s-0029-1216898.
43. Surmont, R.; Verniest, G.; Kimpe, N. de. Short synthesis of the seed germination inhibitor 3,4,5-trimethyl-2(5*H*)-furanone. *J. Org. Chem.* **2010**, *75*, 5750–5753, doi:10.1021/jo1010476.
44. Sharma, V.; Kelly, G.T.; Watanabe, C.M.H. Exploration of the molecular origin of the azinomycin epoxide: timing of the biosynthesis revealed. *Org. Lett.* **2008**, *10*, 4815–4818, doi:10.1021/ol8018852.
127. Bardili, B.; Marschall-Weyerstahl, H.; Weyerstahl, P. Bildung und Reaktivität von hydroxysubstituierten γ- und δ-Lactonen. *Liebigs Ann. Chem.* **1985**, *1985*, 275–300, doi:10.1002/jlac.198519850206.
